# Design, Synthesis, Biological Evaluation and Molecular Docking of Novel F-18-Labeled Focal Adhesion Kinase Inhibitors as Potential Tumor Radiotracers

**DOI:** 10.3390/molecules29061224

**Published:** 2024-03-08

**Authors:** Hailong Yang, Ye Li, Huaju Liang, Chun Cui, Lu Gan, Huabei Zhang

**Affiliations:** Key Laboratory of Radiopharmaceuticals of Ministry of Education, College of Chemistry, Beijing Normal University, No. 19 Xinjiekouwai Street, Haidian District, Beijing 100875, China

**Keywords:** FAK, inhibitor, radiotracer, F-18 label, molecular docking

## Abstract

Tumor diagnosis, especially at the early stages, holds immense significance. Focal adhesion kinase (FAK) is often highly expressed across various types of tumors, making it a promising target for both therapy and diagnosis. In this study, seven novel inhibitors were designed and synthesized. The inhibitory activity of these compounds against FAK was notably potent, with an IC_50_ range of 1.27–1968 nM. In particular, compounds **7a** and **7c**, with IC_50_ values of 5.59 nM and 1.27 nM, respectively, were radiolabeled with F-18 and then evaluated with S-180 tumor-bearing mice. Subsequently, they exhibited moderate-to-high tumor uptake values, with [^18^F]**7a** showing 1.39 ± 0.30%ID/g at 60 min post injection and [^18^F]**7c** demonstrating 6.58 ± 0.46%ID/g at 30 min post injection. In addition, the results from docking studies revealed the binding specifics of the studied compounds. Overall, these findings hold the potential to offer valuable guidance for enhancing the development of radiotracers and enzyme inhibitors.

## 1. Introduction

**Figure 1:**
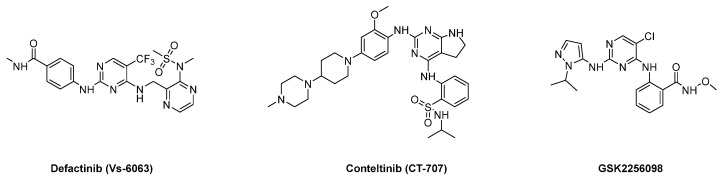
The clinical phase II molecules.

The new case numbers for cancer incidence and associated death are still at high levels, especially for cancers of the lungs, liver, prostate and breast [1,2,3,4]. Focal adhesion kinase (FAK), located both in tumor cells and in the tumor microenvironment, is a non-receptor tyrosine kinase that is overexpressed in most types of cancers and plays an important role in tumor survival, angiogenesis and metastasis [5,6,7], making it a consolidated drug design target for oncological therapy and diagnosis. Most studies have focused on the development of therapeutic drug developments targeting FAK, such as defactinib (VS-6063), conteltinib (CT-707) and GSK2256098, all of which have been investigated in clinical phase II trails (Figure 1). There are also other clinical Phase I and preclinical candidates, including follow-up drug combination studies [8,9].

**Figure 2:**
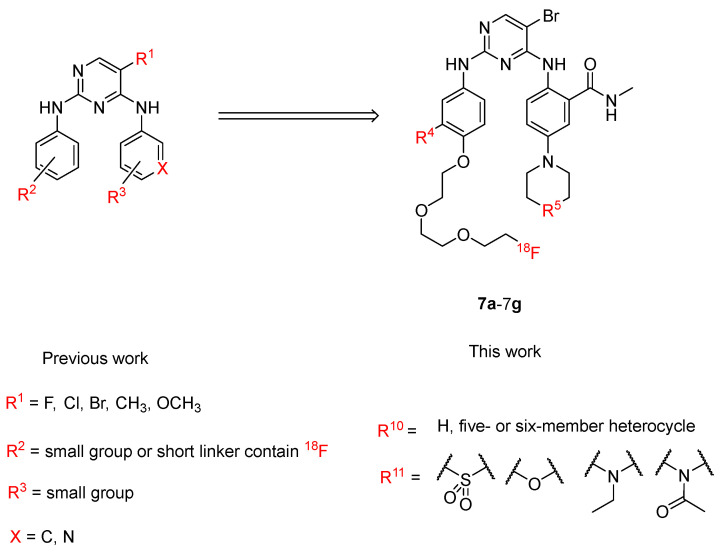
Rational design of radiotracers targeting FAK **7a**–**7g**.

As is well known, the early diagnosis of cancer holds significant value for prolonging the survival time of patients. Positron diagnostic drugs such as F-18-labeled radiotracers are the most sensitive molecules in the field of clinical imaging for tumor diagnosis. However, the target of the radiotracer being non-specific to tumor and tissue exposes the widely known and used ^18^F-FDG to fatal drawbacks such as false-positive results in inflammation and infection or false-negative results in slow-growing tumors. The present study group has been committed to developing novel F-18-labeled radiotracers targeting the tumor-specific FAK, and we have conducted considerable research to seek molecules with high FAK affinity and the proper nature for diagnosis [10,11,12]. However, there is still room for the improvement in the inhibitory activity targeting FAK. Moreover, other features should also be further optimized. As shown in Figure 2, the 2-position of the amidine has been extensively discussed, with PEG linker-substituted aniline being validated to enhance hydrophilicity and provide the F-18-labeled position. Regarding the m-position of the arylamine, CH_3_, OCH_3_, and PEG groups have been previously investigated, with further research in this area to be conducted. In contrast, the 4-position of the pyrimidine, particularly the para-position of the arylamine, has received less academic attention. This might because this position is deeper within the protein, and larger substituted groups could potentially impose a negative impact on the interaction between the molecule and the FAK protein. Through analyzing the protein conformation, a deep pocket large enough to accommodate a six-member circle was found. This concept was first proposed for the whole field of the drug design targeting FAK, including the development of the inhibitors and PROTACs [13,14]. Encouraged by this idea, seven novel FAK inhibitors were designed and synthesized in this study. Their diaminopyrimidine cores guaranteed the formation of the key hydrogen bonds between the molecules and the FAK protein, which was similar to those of clinical molecules. Meanwhile, the difference was that the chemical space of the deep binding sites was extended to explore the effects on the kinase inhibitory and drug properties for biodistribution in vivo.

## 2. Results

### 2.1. Synthesis

**Scheme 1:**
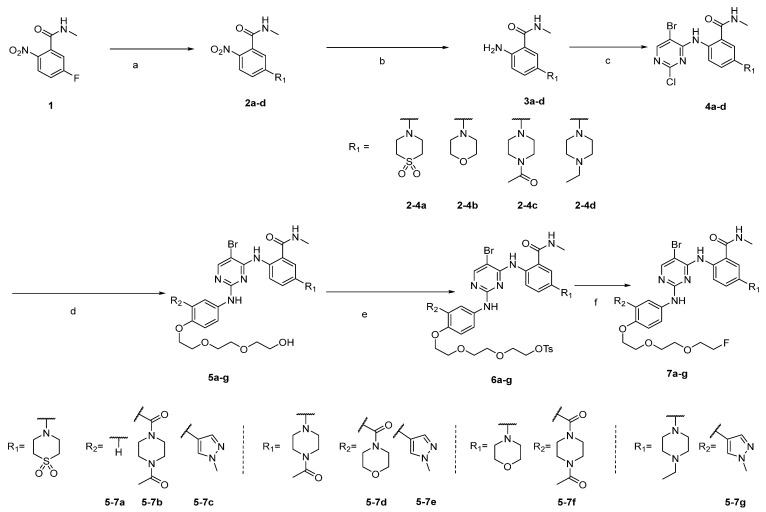
Synthesis of the seven novel derivatives **7**. Reagents and conditions: (**a**) six-membered heterocycle, K_2_CO_3_, ACN, 50 °C; (**b**) Fe powder/NH_4_Cl = 1/1 (*n*/*n*), MeOH/THF/H_2_O = 5/5/2 (*v*/*v*/*v*), 60 °C; (**c**) 5-bromo-2,4-dichloropyrimidine, K_2_CO_3_, DMF, 50 °C; (**d**) corresponding anilines **12** and **14**, TsOH, 1,4-dioxane, 80 °C; (**e**) TsCl, TEA, r.t., DCM; (**f**) TBAF, THF, 50 °C.

Scheme 1 reports the synthesis routes of new derivatives **7a**–**7g**. The six-membered heterocycles (morpholine or thiomorpholine 1,1-dioxide) were introduced to the structure by reacting with the 5-fluoro-*N*-methyl-2-nitrobenzamide at 50 °C. The nitro groups of **2** were then reduced to the amino group with iron filings in the presence of ammonium chloride to obtain **3**. The key intermediate **4** was produced from **3** via a nucleophilic substitution at the 4-position of 5-bromo-2,4-dichloropyrimidine and then treated with different substituted aryl amines (**12** and **14**) to produce derivative **5** using TsOH as a catalyst. It should be noted that the silicon protective group was removed at the same time. This was because deprotection can also be implemented under faintly acidic conditions [15]. The hydroxyl groups were activated by TsCl and substituted by fluoride ions to obtain the labelling precursors and the stable standards. In this way, the two structures were obtained simultaneously, which can also be considered the advantage of this synthesis route.

**Scheme 2:**
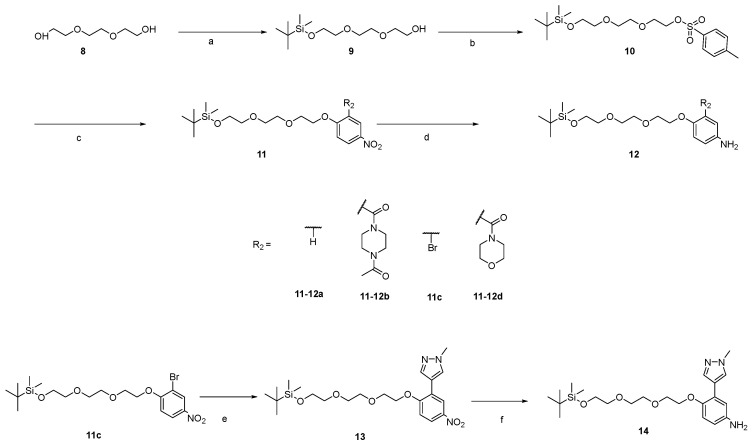
Synthesis of intermediates **8**–**14**. Reagents and conditions: (**a**) TBSCl, imidazole, DCM, 0~r.t.; (**b**) TsCl, TEA, DCM, r.t.; (**c**) corresponding p-nitrophenol, K_2_CO_3_, DMF, 70 °C; (**d**) Fe powder/NH_4_Cl = 1/1 (*n*/*n*), MeOH/THF/H_2_O = 5/5/2 (*v*/*v*/*v*), 60 °C; (**e**) 1-methyl-4-pyrazole boronic acid pinacol ester, PdCl_2_(dppf)Cl_2_CH_2_, Cs_2_CO_3_, 1,4-dioxane, H_2_O, 100 °C; (**f**) Fe powder/NH_4_Cl = 1:1 (*n/n*), MeOH/THF/H_2_O = 5/5/2 (*v*/*v*/*v*), 60 °C.

Scheme 2 presents the synthesis routes of the key intermediates **12a**, **12b**, **12d** and **14**. One oxhydryl of the PEG linker was protected by TBS, and the other was activated by TsCl to obtained **10**. Then, **10** was introduced to the substituted p-notrophenol through a nucleophilic substitution to obtain **11**. Next, **11** was reduced to **12a**, **12b** and **12d**. As to **14**, the Suzuki reaction was conducted after the formation of the new ester bond (**11c**), rather than before, as we have previously tried both strategies. The phenolic hydroxyl group may have a negative effect on the Suzuki reaction.

### 2.2. Radiolabeling and Stability Experiment

**Scheme 3:**
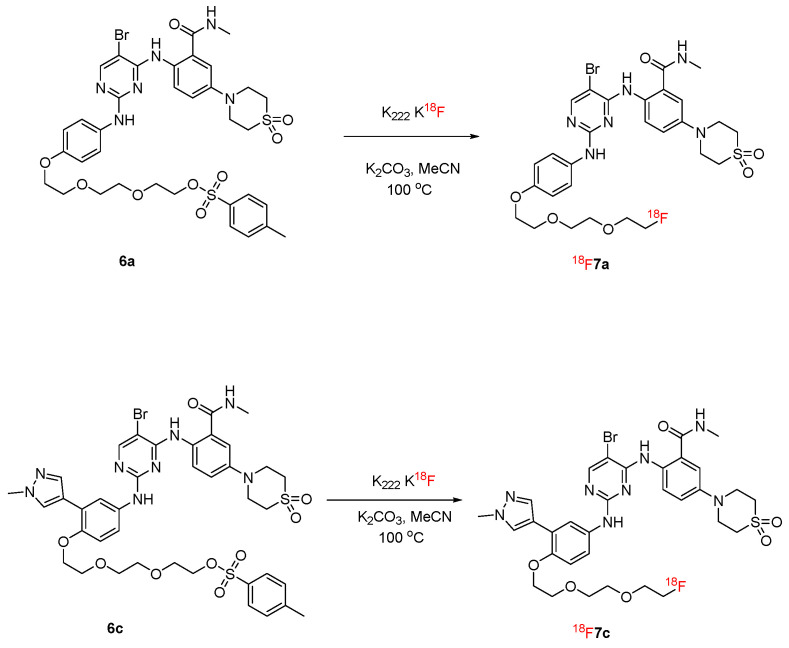
Method of radiolabeling: k_222_K^18^F, K_2_CO_3_, MeCN, 100 °C.

**Figure 3:**
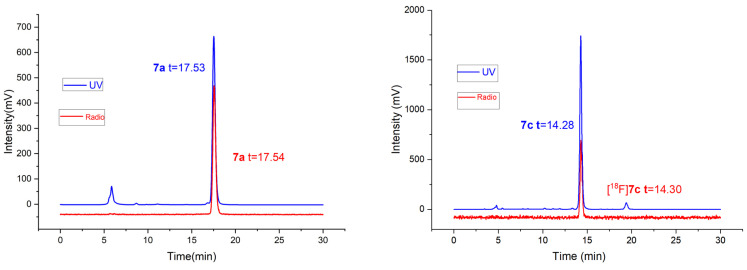
Co-injection of radiotracers with standards.

As shown in Scheme 3, the labeling produced in one step was implemented to produce the [^18^F]**7a** and [^18^F]**7c**. The free ^18^F^−^ ions were obtained using Kryptofix 2.2.2. and potassium carbonate, and then heated to 100 °C in the presence of the labeling precursor for 20 min. The overall radiochemical yields were 15.43% and 17.28%, respectively. After being purified, the two radiotracers presented a high radiochemical purity (>95%). To ensure the correctness of the radioactive products, [^18^F]**7a** and [^18^F]**7c** were co-injected with stable **7a** and **7c**, respectively. The retention period of HPLC was within acceptable error levels, as depicted in Figure 3.

**Figure 4:**
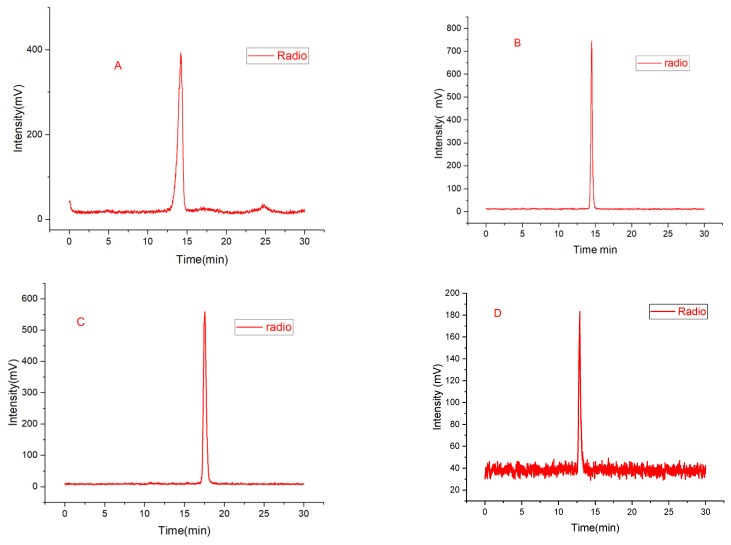
HPLC chromatograms of [^18^F]**7c** and [^18^F]**7a** in the murine serum and saline. (**A**) **7c** in saline, 37 °C, 2 h; (**B**) **7c** in murine serum, 37 °C, 2 h. (**C**) **7a** in Saline, 37 °C, 2 h; (**D**) **7a**, in murine serum, 37 °C, 2 h.

Furthermore, the stability of the two radiotracers in murine serum and saline was studied. As shown in Figure 4, the radiochemical purities were all greater than 95%, indicating their high in vitro stability.

### 2.3. Experiment on Octanol/Water Partition Coefficient Determination

The partition coefficients of the radiotracers [^18^F]**7a** and [^18^F]**7c** were determined to evaluate the octanol/water partition properties. The logD_7.4_s of the two radiotracers were 0.96 and 0.74, respectively, which might be attributable to the possession of the sulfonyl and the pyrazol groups.

### 2.4. FAK Inhibitory Assay

The inhibitory activities of the **7a**–**7c** against FAK were tested using a homogeneous time-resolved fluorescence methodology-based kinase assay. To ensure accuracy, VS-6063 was taken as a positive control under the same conditions. As shown in Table 1, all of the novel compounds demonstrated enzyme inhibition values (IC_50_) in the range of 1.27~1968 nM. Among them, the IC_50_s of **7a** and **7c** were less than 10 nmol (5.59 and 1.27 nM, respectively), which were compared to that of VS-6063 (1.26 nM). The 5-bromo pyrimidine was previously demonstrated to be an attractive core for developing a promising FAK radiotracer. Thus, some pharmacophores containing a hydroxyl group were introduced here to improve the hydrophilicity and the FAK inhibition. It could be clearly observed that the inhibitors containing sulfonyl groups and morpholine showed better inhibition compared to others (IC_50_ of 1.27, 5.59, 10.02, and 24.6 nM vs. 643, 1238, and 1968 nM). This could be attributed to the six-membered aliphatic cyclic structure, which might facilitate suitable penetration into the protein pocket. Interestingly, when comparing **7f** and **7b** (IC_50_: 10.02 vs. 24.6 nM), the morpholine moiety also significantly contributed to the enhanced activity. This could stem from the favorable conformation achieved when the morpholine was positioned at the 4-position of the diaminopyrimidine, while the acetylpiperazine was at the 2-position. To further increase the hydrophilicity, the PEG linker was chosen to form ether with the aniline, which also maintained the high inhibition. To this end, compounds **7a** and **7c**, with high inhibition and good hydrophilicity were chosen for biodistribution studies in S-180 tumor-bearing Kunming mice.

### 2.5. Molecular Docking

**Figure 5:**
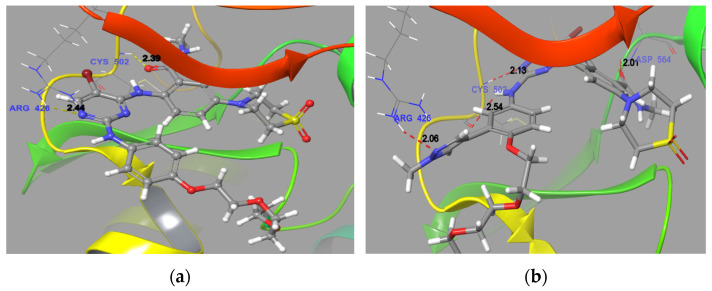
Docking of **7a** ((**a**), IC_50_: 5.59 nM) or **7c** ((**b**), IC_50_: 1.27 nM) with FAK (the crystal structure of FAK was based on PDB code 3BZ3).

Molecular docking studies were conducted to further clarify the binding details between the radiotracers and the FAK protein. As described above, all seven of the new seven pyrimidine derivatives were successfully docked into the FAK protein pocket. Compounds **7a** and **7c** were taken as examples to show the details in Figure 5. Two strong hydrogen bonds were formed between CYS502 and **7c**, with one of the pyrimidine-N and the arylamine-NH acting as H-bond acceptors and H-bond donors, respectively (distance: 2.13 and 2.54 Å, respectively). Meanwhile, amide-O acted as a H-bond acceptor for the NH of ASP564 (distance: 2.01 Å). In addition, **7c** could form one more hydrogen bond between ARG436 and the pyrazole group compared to **7a** (distance: 2.06 Å). Interestingly, **7a** could form only two hydrogen bonds with CYS502 and ARG426. These molecular docking results are consistent with the findings of the FAK inhibitory assays and the biodistribution experiments.

### 2.6. Biodistributions of [^18^F]***7a*** and [^18^F]***7c*** in S-180 Tumor-Bearing Mice

Table 2 presents the biodistributions of [^18^F]**7a** and [^18^F]**7c**. In general, after injection in mice, the two new F-18-labeled compounds showed increased tumor uptake. The peak uptakes of the two radiotracers were observed at 60 min and 30 min post injection, respectively (1.39 ± 0.30%ID/g and 6.58 ± 0.46%ID/g, respectively); the tumor/blood and tumor/muscle ratios for [^18^F]**7c** were 2.40 and 1.88, consistent with the FAK inhibitory experiment results, indicating that the [^18^F]**7a** and [^18^F]**7c** were mainly specific to FAK in vivo. Moreover, the two radiotracers showed good retention and moderate elimination in tumor tissue, and this good nature for diagnosis might result from the hydrophilicity (LogD_7.4_: 0.96 and 0.74, respectively). However, possibly because the two radiotracers were mainly metabolized through the liver and lungs, the uptakes of non-targets tissues like liver and lungs were observed to be high, indicating that they were not suited for the diagnoses of liver and lung tumors.

## 3. Discussion

The FAK protein acts as a hub in the complex network of signaling pathways [16]. Thus, as described above, many researchers have developed therapeutic drugs targeting FAK, such as inhibitors and PROTACs. This also explains the frequent presence of FAK inhibitors in drug combination projects. While remarkable successes have been achieved, none of the candidates have entered the market yet. Concerning the characteristics of FAK as a drug target and the advantages of nuclear scintigraphy techniques, as well as the drawbacks of ^18^F-FDG [17], the research attention of the present study was shifted from therapy to diagnosis targeting FAK.

A total of seven new inhibitors were successfully synthesized. The results of the kinase inhibition assays demonstrated the feasibility of the design strategy described above. On the whole, thiomorpholine 1,1-dioxides and morpholine were suitable for adding to the o-position of the aniline, for the relevant FAK inhibitory were better than others (1.27~24.6 vs. 643~1968 nM). Additionally, one of these molecules (**7c**) possessed essentially the same inhibitory activity targeting FAK as that of the VS-6063 (IC_50_: 1.27 vs. 1.26 nmol). The molecular docking research also demonstrated the same result: **7c** could form two more H-bonds than **7a**. In the future, we can design more novel inhibitors and other related molecules possessing thiomorpholine 1,1-dioxides targeting FAK.

The uptake of the two F-18-labeled molecules in the tumor tissue was obvious, especially for **7c**, and the ID%/g reached 6.58 ± 0.46 at 30 min post injection. This was due to the high FAK affinity of those molecules, indicating the targeting and selection of FAK. Furthermore, the retention and the elimination were acceptable for diagnosis. Interestingly, these two radiotracers ([^18^F]**7a** and [^18^F]**7c**) might undergo significant metabolism through the liver and lungs, as the ID%/g values of these two tissues were high. Although not fit for liver and lung cancer diagnosis, they could still be used for other cancers, e.g., brain, breast, prostate, ovarian, etc. [18,19,20]. Further studies designing radiotracers with intracellular targets should prioritize modulating the logP to minimize the uptake in non-target issues. Nevertheless, the findings of this work will still provide valuable insights that are relevant in this field.

## 4. Materials and Methods

### 4.1. Synthesis

The general organic synthesis route is shown in Scheme 1 and Scheme 2. All the analytical-grade chemical regents used in this study were purchased from commercial sources and used without further purification. Nuclear magnetic resonance spectroscopy (NMR) was performed on a JEOL spectrometer (600 and 400 MHz). Tetramethylsilane (TMS) was used as an internal standard, and chemical shifts are given in (δ) for ^1^H NMR and ^13^C NMR. The mass spectra were recorded on a Waters Quattro Micro Quadrupole Mass Spectrometer.

*2,2,3,3-tetramethyl-4,7,10-trioxa-3-siladodecan-12-ol* (**9**). To a stirring solution of triethylene glycol (10.00 g, 66.59 mmol, 1.00 eq) and imidazole (2.72 g, 39.95 mmol, 0.60 eq) in DCM (150 mL), a solution of t-Butyldimethylsilyl chloride (5.02 g, 33.30 mmol, 0.50 eq) in DCM (50 mL) was added slowly at 0 °C. The temperature was allowed to cool to r.t., and the solution then reacted overnight. After the reaction was completed, the mixture was extracted using H_2_O and DCM. The organic phase was collected and concentrated under medium pressure. The crude product was purified with a flash silica gel column (petroleum ether/ethyl acetate = 3:1 to 1:1) to produce **9** (11.62 g, yellow solid, yield 66.10%). ^1^H NMR (600 MHz, Chloroform-*d*) δ 3.77–3.71 (m, 2H), 3.71–3.66 (m, 2H), 3.59–3.55 (m, 2H), 3.55–3.50 (m, 2H), 2.84–2.67 (m, 1H), 0.85 (d, *J* = 1.6 Hz, 9H), 0.04–0.01 (m, 6H). ^13^C NMR (101 MHz, Chloroform-*d*) δ 72.71, 72.60, 70.82, 70.49, 62.73, 61.75, 25.96, 18.40, −5.26. ESI-MS *m*/*z* 287.1626 [M + Na]^+^.
*2,2,3,3-tetramethyl-4,7,10-trioxa-3-siladodecan-12-yl 4-methylbenzenesulfonate* (**10**). To a stirring solution of **10** (10.00 g, 37.73 mmol, 1.00 eq) and triethylamine (5.72 g, 56.60 mmol, 1.5 eq) in DCM (100 mL), a solution of p-Toluenesulfonyl chloride (8.64 g, 45.24 mmol, 1.2 eq) was added. The mixture was then stirred at room temperature overnight. The reactant solution was concentrated and purified with a flash silica gel column (petroleum ether/ethyl acetate = 10:1 to 3:1) to produce **10** (13.40 g, yellow solid, yield 91.63%). ^1^H NMR (600 MHz, Chloroform-*d*) δ 7.78 (d, *J* = 8.3 Hz, 2H), 7.32 (d, *J* = 7.7 Hz, 2H), 4.20–4.12 (m, 2H), 3.73–3.71 (m, 2H), 3.71–3.64 (m, 2H), 3.58–3.54 (m, 4H), 3.50 (t, *J* = 5.4 Hz, 2H), 2.43 (s, 3H), 0.87 (s, 9H), 0.04 (s, 6H). ^13^C NMR (101 MHz, Chloroform-*d*) δ 144.84, 133.01, 129.87, 128.03, 72.73, 70.82, 70.70, 69.31, 68.71, 62.74, 25.97, 25.74, 21.68, 18.39, −5.23. ESI-MS *m*/*z* 411.1654 [M + Na]^+^.
*2,2,3,3-tetramethyl-12-(4-nitrophenoxy)-4,7,10-trioxa-3-siladodecane* (**11a**). A mixture of **10** (500 mg, 1.29 mmol, 1.00 eq), 4-nitrophenol (269 mg, 1.94 mmol, 1.50 eq), potassium carbonate (359 mg, 2.58 mmol, 2.0 eq) and DMF (10 mL) was stirred at 70 °C overnight [21]. The reaction system was cooled to r.t. and extracted using H_2_O (100 mL) and ethyl acetate (100 mL). The organic phase was combined, washed with brine, dried by anhydrous sodium sulfate and concentrated under medium pressure to obtain **11a** (372 mg, yellow solid, 74.90%). The crude product was used for the next step without further purification. ^1^H NMR (600 MHz, Chloroform-*d*) δ 8.19–8.14 (m, 2H), 6.98–6.94 (m, 2H), 4.23–4.18 (m, 2H), 3.90–3.87 (m, 2H), 3.78–3.73 (m, 2H), 3.72–3.69 (m, 2H), 3.69–3.66 (m, 2H), 3.57–3.53 (m, 2H), 0.87 (d, *J* = 3.6 Hz, 9H), 0.04 (d, *J* = 3.5 Hz, 6H). ^13^C NMR (151 MHz, Chloroform-*d*) δ 163.96, 141.61, 125.88, 125.86, 114.64, 72.77, 71.02, 70.81, 69.41, 68.29, 62.76, 25.94, 18.37, −5.24. ESI-MS *m*/*z* 424.3035 [M + Na]^+^.
*4-((2,2,3,3-tetramethyl-4,7,10-trioxa-3-siladodecan-12-yl)oxy)aniline* (**12a**). To a mixture solvent of THF (5 mL), MeOH (5 mL) and H_2_O (2 mL), **11a** (300 mg, 0.78 mmol, 1.00 eq), iron powder (218 mg, 3.89 mmol, 5.00 eq) and ammonium chloride (210 mg, 3.89 mmol, 5.00 eq) were added [10]. The mixture was stirred overnight at 70 °C. After the reaction was completed, the mixture was filtered and washed with methanol (3 × 10 mL). The organic phase was collected, concentrated under reduced pressure, and purified with a flash silica gel column (petroleum ether/ethyl acetate = 5:1 to 1:1) to produce **12a** (230 mg, brown oil, 83.06%). ^1^H NMR (600 MHz, Chloroform-*d*) *δ* 6.76 (d, *J* = 8.1 Hz, 2H), 6.63 (d, *J* = 8.2 Hz, 2H), 4.09–4.02 (m, 2H), 3.85–3.80 (m, 2H), 3.76 (dt, *J* = 5.6, 2.8 Hz, 2H), 3.72–3.63 (m, 4H), 3.59–3.53 (m, 2H), 0.89 (s, 9H), 0.06 (s, 6H). ^13^C NMR (101 MHz, Chloroform-*d*) δ 151.74, 139.93, 116.12, 115.64, 72.48, 70.61, 70.56, 69.71, 67.91, 62.50, 25.73, 18.17, −5.47. ESI-MS *m*/*z* 356.2208 [M + H]^+^.
*5-(1,1-dioxidothiomorpholino)-N-methyl-2-nitrobenzamide* (**2a**). The method described for **2b** was used to obtain **2a**. ^1^H NMR (600 MHz, DMSO-*d*_6_) δ 8.36 (q, *J* = 4.7 Hz, 1H), 7.98 (d, J = 9.3 Hz, 1H), 7.15 (dd, *J* = 9.3, 2.9 Hz, 1H), 7.02 (d, *J* = 2.9 Hz, 1H), 4.03–3.98 (m, 4H), 3.19–3.13 (m, 4H), 2.75 (d, *J* = 4.6 Hz, 3H). ^13^C NMR (101 MHz, DMSO-*d*_6_) δ 167.23, 151.66, 136.72, 136.46, 127.32, 114.17, 113.64, 50.81, 46.00, 26.58. ESI-MS *m*/*z* 314.0780 [M + H]^+^.
*2-amino-5-(1,1-dioxidothiomorpholino)-N-methylbenzamide* (**3a**). The method described for **12a** was used to obtain **3a**. ^1^H NMR (400 MHz, DMSO-*d*_6_) *δ* 8.19 (d, *J* = 4.7 Hz, 1H), 7.07 (d, *J* = 2.8 Hz, 1H), 6.96 (dd, *J* = 8.8, 2.8 Hz, 1H), 6.64 (d, *J* = 8.8 Hz, 1H), 6.02 (s, 2H), 3.61–3.40 (m, 4H), 3.23–3.08 (m, 4H), 2.72 (d, *J* = 4.5 Hz, 3H). ^13^C NMR (100 MHz, DMSO-*d*_6_, *δ* ppm): 169.58, 139.57, 123.47, 118.49, 117.31, 50.82, 49.47, 26.43. ESI-MS *m*/*z* 284.1038 [M + H]^+^.
*2-((5-bromo-2-chloropyrimidin-4-yl)amino)-5-(1,1-dioxidothiomorpholino)-N-methylbenzamide* (**4a**). The method described in **4b** was used to obtain **4a**. ^1^H NMR (600 MHz, DMSO-*d*_6_) *δ* 11.52 (s, 1H), 8.83–8.79 (m, 1H), 8.47 (s, 1H), 8.29 (d, *J* = 9.0 Hz, 1H), 7.30–7.25 (m, 2H), 3.84 (t, *J* = 5.3 Hz, 4H), 3.17 (t, *J* = 5.3 Hz, 4H), 2.81 (d, *J* = 4.5 Hz, 3H). ^13^C NMR (101 MHz, DMSO-*d*_6_) δ 169.15, 158.35, 158.09, 157.35, 143.87, 130.34, 123.69, 123.18, 119.40, 115.28, 105.08, 50.32, 47.24, 26.80. ESI-MS *m*/*z* 473.9926 [M + H]^+^.
*2-((5-bromo-2-((4-(2-(2-(2-hydroxyethoxy)ethoxy)ethoxy)phenyl)amino)pyrimidin-4-yl)amino)-5-(1,1-dioxidothiomorpholino)-N-methylbenzamide* (**5a**). A solution of **4a** (133 mg, 0.28 mmol, 1.00 eq), **12a** (120 mg, 0.34 mmol, 1.20 eq) and 4-Toluenesulfonic acid (7 mg, 0.03 mmol, 0.10 eq) in 1,4-dioxane was stirred at 80 °C for 48 h. After the reaction was completed, the mixture was purified with a flash silica gel column (dichloromethane/methanol = 20:1 to 10:1) to produce **5a** (110 mg, brown solid, 57.77%). ^1^H NMR (400 MHz, DMSO-*d*_6_) *δ* 10.80 (s, 1H), 9.20 (s, 1H), 8.71 (d, *J* = 4.6 Hz, 1H), 8.39 (d, *J* = 8.8 Hz, 1H), 8.18 (s, 1H), 7.51 (d, *J* = 8.7 Hz, 2H), 7.24 (d, *J* = 2.8 Hz, 1H), 7.17 (dd, *J* = 9.2, 2.9 Hz, 1H), 6.94–6.79 (m, 2H), 4.59 (t, *J* = 5.5 Hz, 1H), 4.05 (t, *J* = 4.6 Hz, 2H), 3.85–3.79 (m, 4H), 3.73 (t, *J* = 4.7 Hz, 2H), 3.61–3.57 (m, 2H), 3.57–3.53 (m, 2H), 3.51–3.47 (m, 2H), 3.45–3.41 (m, 2H), 3.21–3.16 (m, 4H), 2.80 (d, *J* = 4.5 Hz, 3H). ^13^C NMR (100 MHz, DMSO-*d*_6_, δ ppm): 168.79, 158.47, 157.14, 155.67, 153.70, 142.70, 133.43, 131.24, 123.54, 123.06, 121.53, 118.90, 115.02, 114.34, 93.38, 72.39, 70.00, 69.82, 69.10, 67.34, 60.26, 49.88, 47.16, 26.26. ESI-MS *m*/*z* 679.1574 [M + H]^+^.
*2-(2-(2-(4-((5-bromo-4-((4-(1,1-dioxidothiomorpholino)-2-(methylcarbamoyl)phenyl)amino)pyrimidin-2-yl)amino)phenoxy)ethoxy)ethoxy)ethyl 4-methylbenzenesulfonate* (**6a**). The method described for **10** was used to produce **6a** (77 mg, white solid, 62.90% yield). ^1^HNMR (600 MHz, DMSO-*d*_6_) δ 10.82 (s, 1H), 9.21 (s, 1H), 8.72 (q, *J* = 4.8 Hz, 1H), 8.46–8.36 (m, 1H), 8.18 (s, 1H), 7.81–7.75 (m, 2H), 7.51 (d, *J* = 8.6 Hz, 2H), 7.46 (d, *J* = 8.1 Hz, 2H), 7.24 (d, *J* = 3.0 Hz, 1H), 7.18–7.12 (m, 1H), 6.89–6.83 (m, 2H), 4.12–4.09 (m, 2H), 4.05–4.01 (m, 2H), 3.83–3.79 (m, 4H), 3.72–3.68 (m, 2H), 3.59–3.56 (m, 2H), 3.55–3.51 (m, 2H), 3.49–3.46 (m, 2H), 3.21–3.16 (m, 4H), 2.80 (d, *J* = 4.7 Hz, 3H), 2.39 (s, 3H). ^13^C NMR (101 MHz, DMSO-*d*_6_) δ 169.28, 158.91, 157.58, 156.15, 154.15, 145.44, 143.13, 133.92, 132.91, 131.72, 130.67, 128.17, 124.00, 123.48, 121.95, 119.35, 115.51, 114.79, 93.87, 70.51, 70.33, 70.25, 69.59, 68.45, 67.75, 50.33, 47.61, 26.76, 21.61. ESI-MS *m*/*z* 833.1060 [M + H]^+^.
*2-((5-bromo-2-((4-(2-(2-(2-fluoroethoxy)ethoxy)ethoxy)phenyl)amino)pyrimidin-4-yl)amino)-5-(1,1-dioxidothiomorpholino)-N-methylbenzamide* (**7a**). To a solution of **6a** (50 mg, 0.06 mmol, 1.00 eq) in THF (1 mL), tetrabutylammonium fluoride (1 mol/L in THF, 0.30 mL, 5.00 eq) was added slowly, and then reacted for 2 h at 50 °C. After the reaction completed, the mixture was extracted using ethyl acetate and water. The organic phase was combined, washed with brine, dried using anhydrous sodium sulfate, concentrated under medium pressure and purified with a flash silica gel column (dichloromethane/methanol = 20:1 to 10:1) to produce **7a** (28 mg, white solid, 68.53%) [22]. *R*_f_ (MeOH/DCM = 1/10) = 0.58. ^1^H NMR (600 MHz, DMSO-*d*_6_) δ 10.83 (s, 1H), 9.23 (s, 1H), 8.71 (q, *J* = 4.6 Hz, 1H), 8.38 (s, 1H), 8.18 (s, 1H), 7.50 (d, *J* = 8.4 Hz, 2H), 7.24 (d, *J* = 2.9 Hz, 1H), 7.16 (dd, *J* = 9.2, 3.0 Hz, 1H), 6.87 (d, *J* = 8.6 Hz, 2H), 4.52 (dt, *J* = 48.0, 4.0 Hz, 2H), 4.05 (t, *J* = 4.6 Hz, 2H), 3.82 (t, *J* = 5.1 Hz, 4H), 3.74 (t, *J* = 4.6 Hz, 2H), 3.70–3.67 (m, 1H), 3.64–3.62 (m, 1H), 3.62–3.58 (m, 4H), 3.21–3.16 (m, 4H), 2.80 (d, *J* = 4.5 Hz, 3H). ^13^C NMR (100 MHz, DMSO-*d*_6_, δ ppm): 169.27, 158.95, 157.62, 156.16, 154.16, 143.14, 133.95, 131.75, 124.00, 123.48, 121.98, 119.35, 115.55, 114.81, 93.85, 84.40, 82.75, 70.45, 70.38, 70.33, 70.14, 69.61, 67.80, 50.37, 47.64, 26.74. The ESI-HRMS *m*/*z* calculated for C_28_H_35_BrFN_6_O_6_S^+^ 681.1501 was found to be 681.1498. IR for **7a** (KBr, cm^−1^): 3230 (vw), 2860 (vw), 1570 (m), 1510 (s), 1420 (2), 1320 (w), 1280 (m), 1230 (m), 1180 (m), 1120 (w), 1010 (w), 928 (w), 865 (w), 773 (m), 539 (m).
*morpholino(5-nitro-2-((2,2,3,3-tetramethyl-4,7,10-trioxa-3-siladodecan-12-yl)oxy)phenyl)methanone* (**11d**). The method described for **11a** was used to produce **11d** (yellow solid, 71.23%). ^1^H NMR (600 MHz, Chloroform-*d*) δ 8.25 (dd, *J* = 9.1, 2.8 Hz, 1H), 8.19 (d, *J* = 2.8 Hz, 1H), 7.01 (d, *J* = 9.1 Hz, 1H), 4.38–4.29 (m, 1H), 4.25–4.18 (m, 1H), 3.95–3.87 (m, 1H), 3.86–3.83 (m, 1H), 3.82–3.78 (m, 2H), 3.77–3.74 (m, 4H), 3.73–3.70 (m, 1H), 3.69–3.64 (m, 4H), 3.60–3.56 (m, 1H), 3.54 (t, *J* = 5.2 Hz, 2H), 3.40–3.29 (m, 1H), 3.27–3.15 (m, 1H), 0.88 (s, 9H), 0.05 (s, 6H). ^13^C NMR (100 MHz, DMSO-*d*_6_, δ ppm): 165.28, 159.42, 141.78, 126.74, 124.61, 111.78, 72.83, 71.05, 69.28, 68.95, 66.80, 62.76, 61.83, 60.48, 47.28, 42.40, 25.99, 25.72, −3.51, −5.20. ESI-MS *m*/*z* 499.2460 [M + H]^+^.
*(5-amino-2-((2,2,3,3-tetramethyl-4,7,10-trioxa-3-siladodecan-12-yl)oxy)phenyl)(morpholino)methanone* (**12d**). The method described for **12a** was used to produce **12d** (brown oil, 85.21% yield). ^1^H NMR (600 MHz, Chloroform-*d*) δ 6.76–6.72 (m, 1H), 6.69–6.65 (m, 1H), 6.64–6.62 (m, 1H), 4.15–4.06 (m, 1H), 4.03–3.97 (m, 1H), 3.83–3.79 (m, 1H), 3.79–3.75 (m, 4H), 3.74–3.70 (m, 4H), 3.67–3.64 (m, 4H), 3.58–3.50 (m, 3H), 3.42–3.35 (m, 1H), 3.26–3.16 (m, 1H), 0.89–0.86 (m, 9H), 0.07–0.03 (m, 6H). ^13^C NMR (151 MHz, Chloroform-*d*) δ 167.81, 147.16, 140.93, 126.68, 117.04, 114.95, 114.57, 72.65, 70.75, 70.68, 69.79, 69.00, 66.91, 66.71, 62.65, 47.24, 42.11, 25.91, 18.32, −5.28. ESI-MS *m*/*z* 469.2664 [M + H]^+^.
*5-(4-acetylpiperazin-1-yl)-2-((5-bromo-2-((4-(2-(2-(2-hydroxyethoxy)ethoxy)ethoxy)-3-(morpholine-4-carbonyl)phenyl)amino)pyrimidin-4-yl)amino)-N-methylbenzamide* (**5d**). The method described for **5a** was used to produce **5d** (white solid, yield 53.18%). ^1^H NMR (600 MHz, DMSO-*d*_6_) δ 9.24 (s, 1H), 9.10 (q, *J* = 4.8 Hz, 1H), 8.68 (s, 1H), 8.18 (s, 1H), 7.83 (d, *J* = 2.7 Hz, 1H), 7.74 (s, 1H), 7.67 (dd, *J* = 9.1, 2.7 Hz, 1H), 7.34–7.24 (m, 1H), 7.20 (d, *J* = 8.7 Hz, 1H), 6.83 (d, *J* = 9.0 Hz, 1H), 4.58 (t, *J* = 5.5 Hz, 1H), 4.10–4.00 (m, 2H), 3.75–3.68 (m, 2H), 3.66–3.61 (m, 4H), 3.61–3.55 (m, 8H), 3.55–3.53 (m, 2H), 3.49–3.47 (m, 2H), 3.41 (t, *J* = 5.1 Hz, 2H), 3.23–3.03 (m, 2H), 2.96–2.91 (m, 2H), 2.87 (t, *J* = 5.2 Hz, 2H), 2.85 (d, *J* = 4.8 Hz, 3H), 2.05 (s, 3H). ^13^C NMR (100 MHz, DMSO-*d*_6_, δ ppm):168.24, 166.35, 166.16, 157.88, 157.25, 156.50, 148.47, 146.21, 134.28, 133.67, 128.70, 126.19, 125.14, 124.97, 120.91, 119.93, 118.36, 112.18, 92.27, 72.16, 69.82, 69.59, 68.82, 67.70, 66.00, 65.84, 60.00, 52.63, 52.07, 46.46, 45.63, 41.40, 40.89, 25.74, 21.03. ESI-MS *m*/*z* 785.2104 [M + H]^+^.
*2-(2-(2-(4-((4-((4-(4-acetylpiperazin-1-yl)-2-(methylcarbamoyl)phenyl)amino)-5-bromopyrimidin-2-yl)amino)-2-(morpholine-4-carbonyl)phenoxy)ethoxy)ethoxy)ethyl 4-methylbenzenesulfonate* (**6d**). The method described for **10** was used to produce **6d** (white solid, yield 57.2%). ^1^H NMR (600 MHz, DMSO-*d*_6_) δ 9.25 (s, 1H), 9.10 (q, *J* = 4.7 Hz, 1H), 8.68 (s, 1H), 8.19 (s, 1H), 7.83 (d, *J* = 2.8 Hz, 1H), 7.77 (d, *J* = 8.2 Hz, 2H), 7.74 (s, 1H), 7.67 (dd, *J* = 9.0, 2.7 Hz, 1H), 7.46 (d, *J* = 8.1 Hz, 2H), 7.26 (s, 1H), 7.19 (d, *J* = 8.7 Hz, 1H), 6.82 (d, *J* = 8.9 Hz, 1H), 4.17–4.08 (m, 2H), 4.03 (t, *J* = 5.3 Hz, 2H), 3.68 (t, *J* = 4.8 Hz, 2H), 3.64–3.60 (m, 4H), 3.60–3.54 (m, 8H), 3.53–3.49 (m, 2H), 3.50–3.45 (m, 2H), 3.21–3.02 (m, 2H), 2.93 (t, *J* = 5.5 Hz, 2H), 2.87 (t, *J* = 5.0 Hz, 2H), 2.84 (d, *J* = 4.7 Hz, 3H), 2.39 (s, 3H), 2.04 (s, 3H).^13^C NMR (151 MHz, DMSO-*d*_6_) δ 168.40, 166.52, 166.34, 158.08, 156.69, 148.64, 146.39, 144.88, 134.48, 133.88, 132.40, 130.11, 128.89, 127.59, 125.33, 125.18, 121.09, 120.11, 118.54, 112.34, 69.94, 69.86, 69.70, 69.00, 67.93, 67.85, 66.16, 66.01, 52.82, 52.27, 46.63, 45.92, 41.57, 41.07, 25.93, 21.22, 21.05. ESI-MS *m*/*z* 939.2494 [M + H]^+^.
*5-(4-acetylpiperazin-1-yl)-2-((5-bromo-2-((4-(2-(2-(2-fluoroethoxy)ethoxy)ethoxy)-3-(morpholine-4-carbonyl)phenyl)amino)pyrimidin-4-yl)amino)-N-methylbenzamide* (**7d**). The method described for **7a** was used to produce **7d** (white solid, yield 65.4%). *R*_f_ (MeOH/DCM = 1/10) = 0.47. ^1^H NMR (600 MHz, DMSO-*d*_6_) δ 9.26 (s, 1H), 9.10 (q, *J* = 4.7 Hz, 1H), 8.70 (s, 1H), 8.19 (s, 1H), 7.83 (d, *J* = 2.7 Hz, 1H), 7.74 (d, *J* = 8.5 Hz, 1H), 7.67 (dd, *J* = 9.0, 2.7 Hz, 1H), 7.26 (s, 1H), 7.20 (d, *J* = 8.7 Hz, 1H), 6.83 (d, *J* = 9.1 Hz, 1H), 4.58–4.53 (m, 1H), 4.52–4.45 (m, 1H), 4.09–4.01 (m, 2H), 3.72 (td, *J* = 5.6, 3.0 Hz, 2H), 3.68–3.66 (m, 1H), 3.65–3.64 (m, 1H), 3.63–3.60 (m, 4H), 3.60–3.55 (m, 8H), 3.46–3.41 (m, 2H), 3.22–3.17 (m, 1H), 3.11–3.05 (m, 1H), 2.93 (t, *J* = 5.3 Hz, 2H), 2.87 (t, *J* = 5.1 Hz, 2H), 2.85 (d, *J* = 4.7 Hz, 3H), 2.05 (s, 3H).^13^C NMR (100 MHz, DMSO-*d*_6_, δ ppm): 168.03, 166.13, 165.94, 167.55, 156.33, 148.29, 146.33, 134.04, 133.39, 128.47, 126.05, 124.92, 124.83, 120.76, 129.73, 128.20, 111.93, 83.45, 81.81, 69.59, 69.45, 69.41, 69.22, 69.63, 67.45, 65.80, 65.64, 52.43, 51.88, 46.25, 45.52, 41.18, 40.67, 25.55, 20.84. The ESI-HRMS *m*/*z* calculated for C_35_H_45_BrFN_8_O_7_^+^ 787.2573 was found to be 787.2573. IR for **7d** (KBr, cm^−1^): 3280 (w), 2850 (m), 1630 (s), 1640 (s), 1490 (m), 1420 (vs), 1240 (s), 1110 (s), 1030 (m), 999 (m), 896 (m), 810 (m), 771 (m), 698 (w), 643 (w), 578 (w), 554 (s), 524 (m).
*N-methyl-5-morpholino-2-nitrobenzamide* (**2b**). To a stirring mixture of 5-fluoro-N-methyl-2-nitrobenzamide (2.0 g, 10.1 mmol, 1.0 eq), K_2_CO_3_ (2.8 g, 20.2 mmol, 2.0 eq) and acetonitrile (20 mL), morpholine (0.97 g, 11.1 mmol, 1.1 eq) was added. Then, the reaction continued to react overnight at 50 °C. After the reaction was completed, the mixture was poured into water, the solid was filtered under a vacuum, washed with water (50 mL, 3 times) and dried naturally to obtained **2b** (yellow solid, 1.9 g, yield 71.2%). ^1^H NMR (600 MHz, DMSO-*d*_6_) δ 8.31 (d, *J* = 4.7 Hz, 1H), 7.99 (d, *J* = 9.3 Hz, 1H), 7.03 (dd, *J* = 9.4, 2.9 Hz, 1H), 6.88 (d, *J* = 2.9 Hz, 1H), 3.71 (t, 4H), 3.41 (t, *J* = 5.0 Hz, 4H), 2.74 (d, *J* = 4.6 Hz, 3H). ^13^C NMR (101 MHz, DMSO-*d*_6_) δ 167.06, 153.90, 136.27, 135.09, 126.70, 112.64, 112.19, 65.74, 46.50, 26.07. ESI-MS *m*/*z* 266.1110 [M + H]^+^.
*2-amino-N-methyl-5-morpholinobenzamide* (**3b**). The method described for **12a** was used to produce **3b** (brown solid, yield 82.1%). ^1^H NMR (600 MHz, Chloroform-*d*) δ 6.99–6.84 (m, 2H), 6.67 (d, *J* = 8.2 Hz, 1H), 6.15 (s, 1H), 3.85 (t, *J* = 4.7 Hz, 4H), 3.01 (s, 4H), 2.96 (d, *J* = 4.9 Hz, 3H). ^13^C NMR (151 MHz, DMSO-*d*_6_) δ 169.26, 143.46, 141.11, 121.69, 117.23, 115.32, 66.13, 50.43, 25.77. ESI-MS *m*/*z* 236.0715 [M + H]^+^.
*2-((5-bromo-2-chloropyrimidin-4-yl)amino)-N-methyl-5-morpholinobenzamide* (**4b**). To a solution of 5-bromo-2,4-dichloropyrimidine (1.0 g, 4.4 mmol, 1.0 eq) in DMF (10 mL), **3b** (1.0 g, 4.4 mmol, 1.0 eq) and K_2_CO_3_ (917 mg, 6.6 mmol, 1.6 eq) were added slowly. The mixture was allowed to react overnight at 50 °C, and then, it was poured into water and filtered. The filter cake was washed with water (50 mL, 3 times) and dried naturally to obtained **4b** (1.3 g, yellow solid, 67.4%) [10]. ^1^H NMR (600 MHz, DMSO-*d*_6_) δ 11.57 (s, 1H), 8.81 (d, *J* = 4.8 Hz, 1H), 8.46 (s, 1H), 8.27 (d, *J* = 9.1 Hz, 1H), 7.26 (d, *J* = 2.9 Hz, 1H), 7.20 (dd, *J* = 9.2, 2.9 Hz, 1H), 3.76 (t, *J* = 4.8 Hz, 4H), 3.17 (t, *J* = 5.8, 3.9 Hz, 4H), 2.80 (d, *J* = 4.5 Hz, 3H). ^13^C NMR (101 MHz, DMSO-*d*_6_) δ 168.73, 157.74, 157.58, 156.82, 146.86, 129.81, 122.72, 122.42, 118.14, 114.14, 104.54, 66.00, 48.43, 26.29.ESI-MS *m*/*z* 426.0280 [M + H]^+^.
*2-((2-((3-(4-acetylpiperazine-1-carbonyl)-4-(2-(2-(2-hydroxyethoxy)ethoxy)ethoxy)phenyl)amino)-5-bromopyrimidin-4-yl)amino)-N-methyl-5-morpholinobenzamide* (**5f**). The method described for **5a** was used to obtain **5f** (white solid, yield 55.7%). ^1^H NMR (600 MHz, DMSO-*d*_6_) δ 10.98 (d, *J* = 12.8 Hz, 1H), 9.32 (d, *J* = 11.3 Hz, 1H), 8.77–8.66 (m, 1H), 8.42 (s, 1H), 8.20 (d, *J* = 4.0 Hz, 1H), 7.64–7.58 (m, 1H), 7.51 (d, *J* = 26.2 Hz, 1H), 7.24–7.19 (m, 1H), 7.12 (dt, *J* = 9.3, 3.6 Hz, 1H), 7.01 (dd, *J* = 9.0, 3.4 Hz, 1H), 4.58–4.55 (m, 1H), 4.17–4.04 (m, 2H), 3.79–3.74 (m, 4H), 3.73–3.68 (m, 2H), 3.57–3.54 (m, 2H), 3.52–3.45 (m, 8H), 3.45–3.37 (m, 6H), 3.15 (t, *J* = 5.0 Hz, 4H), 2.79 (d, *J* = 4.5 Hz, 3H), 2.00 (d, *J* = 40.1 Hz, 3H). ^13^C NMR (101 MHz, DMSO-*d*_6_) δ 169.43, 168.87, 167.05, 158.71, 157.47, 156.15, 149.48, 146.53, 145.97, 138.34, 134.43, 131.73, 128.64, 126.06, 123.27, 119.00, 114.78, 113.27, 94.40, 72.89, 70.48, 70.29, 69.57, 68.46, 66.59, 60.72, 49.32, 26.78, 21.32. ESI-MS *m*/*z* 785.2112 [M + H]^+^.
*2-(2-(2-(2-(4-acetylpiperazine-1-carbonyl)-4-((5-bromo-4-((2-(methylcarbamoyl)-4-morpholinophenyl)amino)pyrimidin-2-yl)amino)phenoxy)ethoxy)ethoxy)ethyl 4-methylbenzenesulfonate* (**6f**). The method described for **6a** was used to obtain **6f** (white solid, yield 74.2%) ^1^H NMR (600 MHz, DMSO-*d*_6_) δ 10.99 (d, *J* = 12.9 Hz, 1H), 9.33 (d, *J* = 11.4 Hz, 1H), 8.83–8.64 (m, 1H), 8.42 (s, 1H), 8.20 (d, *J* = 4.0 Hz, 1H), 7.77 (d, *J* = 8.0 Hz, 2H), 7.64–7.59 (m, 1H), 7.55–7.48 (m, 1H), 7.46 (d, *J* = 7.9 Hz, 2H), 7.22 (s, 1H), 7.15–7.10 (m, 1H), 7.06–6.97 (m, 1H), 4.13–4.05 (m, 4H), 3.78–3.75 (m, 4H), 3.68 (d, *J* = 4.8 Hz, 4H), 3.54 (t, *J* = 4.4 Hz, 4H), 3.51–3.47 (m, 4H), 3.45–3.41 (m, 4H), 3.15 (t, *J* = 4.9 Hz, 4H), 2.79 (d, *J* = 4.4 Hz, 3H), 2.39 (d, *J* = 2.6 Hz, 3H), 1.99 (d, *J* = 41.7 Hz, 3H).^13^C NMR (101 MHz, DMSO-*d*_6_) δ 169.41, 168.86, 167.00, 158.33, 156.20, 149.57, 146.60, 145.44, 134.24, 132.90, 131.62, 130.66, 128.61, 128.16, 126.07, 123.32, 122.81, 122.18, 119.56, 119.22, 118.96, 114.78, 113.22, 94.42, 70.47, 70.32, 70.19, 69.54, 68.44, 66.58, 55.46, 49.28, 26.79, 21.74, 21.60. ESI-MS *m*/*z* 939.2455 [M + H]^+^.
*3-((2-((3-(4-acetylpiperazine-1-carbonyl)-4-(2-(2-(2-fluoroethoxy)ethoxy)ethoxy)phenyl)amino)-5-bromopyrimidin-4-yl)amino)-N-methyl-5-morpholinobenzamide* (**7f**). The method described for **7a** was used to obtain **7f** (whited solid, yield 62.3%). *R*_f_ (MeOH/DCM = 1/10) = 0.42. ^1^H NMR (600 MHz, DMSO-*d*_6_) δ 10.98 (d, *J* = 13.0 Hz, 1H), 9.31 (d, *J* = 11.6 Hz, 1H), 8.78–8.59 (m, 1H), 8.42 (s, 1H), 8.20 (d, *J* = 4.0 Hz, 1H), 7.67–7.56 (m, 1H), 7.51 (d, *J* = 26.8 Hz, 1H), 7.22 (t, *J* = 2.2 Hz, 1H), 7.12 (dt, *J* = 9.1, 3.4 Hz, 1H), 7.01 (dd, *J* = 9.1, 3.9 Hz, 1H), 4.60–4.41 (m, 2H), 4.16–4.05 (m, 2H), 3.77 (t, *J* = 4.8 Hz, 4H), 3.72 (q, *J* = 5.1 Hz, 2H), 3.67–3.62 (m, 2H), 3.62–3.55 (m, 4H), 3.55–3.52 (m, 2H), 3.51–3.38 (m, 4H), 3.28–3.19 (m, 1H), 3.16 (t, *J* = 4.9 Hz, 4H), 3.12–3.00 (m, 1H), 2.79 (d, *J* = 4.5 Hz, 3H), 2.00 (d, *J* = 40.3 Hz, 3H). ^13^C NMR (100 MHz, DMSO-*d*_6_, δ ppm): 169.43, 168.85, 166.97, 158.69, 157.49, 156.16, 149.41, 146.54, 134.49, 131.72, 130.19, 126.13, 123.28, 122.79, 119.24, 119.00, 114.79, 113.33, 94.14, 84.37, 82.72, 70.43, 70.31, 70.12, 69.58, 68.52, 68.43, 66.59, 49.32, 26.77, 21.75. The ESI-HRMS *m*/*z* calculated for C_35_H_45_BrFN_8_O_7_^+^ 787.2573 was found to be 787.2573. IR for **7f** (KBr, cm^−1^): 3270 (m), 2920 (m), 2850 (m), 1550 (m), 1500 (m), 1410 (s), 1240 (s), 1120 (s), 1050 (m), 997 (m), 957 (m), 878 (m), 818 (m), 774 (m), 613 (w), 584 (w), 540 (w), 512 (w).
*(2-bromo-4-nitrophenoxy)-2,2,3,3-tetramethyl-4,7,10-trioxa-3-siladodecane* (**11c**). The method described for **11a** was used to obtain **11c** (yellow solid, yield 77.2%).^1^H NMR (600 MHz, Chloroform-*d*) δ 8.46 (d, *J* = 2.7, 0.7 Hz, 1H), 8.18 (dd, *J* = 9.1, 2.7, 0.8 Hz, 1H), 7.05–6.94 (m, 1H), 4.29 (t, *J* = 4.8 Hz, 2H), 3.99–3.92 (m, 2H), 3.78–3.75 (m, 4H), 3.70–3.68 (m, 2H), 3.57–3.55 (m, 2H), 0.88 (s, 9H), 0.05 (s, 6H). ^13^C NMR (100 MHz, Chloroform-*d*, δ ppm): 160.32, 141.47, 129.02, 124.51, 112.07, 111.78, 72.63, 71.18, 70.71, 69.64, 69.10, 62.62, 25.80, 25.52, −5.39.ESI-MS *m*/*z* 464.1007 [M + H]^+^.
*1-methyl-4-(5-nitro-2-((2,2,3,3-tetramethyl-4,7,10-trioxa-3-siladodecan-12-yl)oxy)phenyl)-1H-pyrazole* (**13**). In a one-neck bottle, **11c** (500 mg, 1.08 mmol), 1-methyl-4-(4,4,5,5-tetramethyl-1,3,2-dioxaborolan-2-yl)-1H-pyrazole (337 mg, 1.62 mmol), 1,1′-Bis (diphenylphosphino) ferrocene-palladium (II) dichloride dichloromethane complex (90 mg, 0.11 mmol), Cs_2_CO_3_ (706.3 mg, 2.16 mmol), 1,4-dioxane (5 mL) and H_2_O (1 mL), the mixture was allowed to react for 16 h under a nitrogen atmosphere. Then, the solvent was removed under a vacuum, and the crude product was purified with a flash silica gel column (dichloromethane/MeOH = 30/1~20/1) to produce **13** (brown oil, 380 mg, 76%) [23]. ^1^H NMR (600 MHz, Chloroform-*d*) δ 8.40 (d, *J* = 2.8 Hz, 1H), 8.07 (ddd, *J* = 9.0, 2.8, 1.1 Hz, 1H), 8.00 (d, *J* = 24.1 Hz, 2H), 6.97 (d, *J* = 9.0 Hz, 1H), 4.31 (t, *J* = 4.7 Hz, 2H), 3.98–3.95 (m, 5H), 3.77–3.73 (m, 4H), 3.73–3.70 (m, 2H), 3.57 (t, *J* = 5.3, 1.1 Hz, 2H), 0.87 (s, 9H), 0.04 (s, 6H). ^13^C NMR (100 MHz, DMSO-*d*_6_, δ ppm): 159.66, 141.81, 138.40, 130.46, 123.05, 122.95, 122.63, 116.84, 111.55, 72.88, 70.98, 70.93, 69.41, 68.22, 62.78, 39.18, 26.00, 24.96, −5.20. ESI-MS *m*/*z* 466.2317 [M + H]^+^.
*3-(1-methyl-1H-pyrazol-4-yl)-4-((2,2,3,3-tetramethyl-4,7,10-trioxa-3-siladodecan-12-yl)oxy)aniline* (**14**). The method described for **12a** was used to obtain **14** (brown oil, yield 78.3%). ^1^H NMR (600 MHz, Chloroform-*d*) δ 7.96 (s, 1H), 7.84 (s, 1H), 6.93 (d, *J* = 2.8 Hz, 1H), 6.77 (d, *J* = 8.6 Hz, 1H), 6.56 (dd, *J* = 8.6, 2.8 Hz, 1H), 4.12–4.07 (m, 2H), 3.92 (s, 3H), 3.87–3.84 (m, 2H), 3.76 (t, *J* = 5.4 Hz, 2H), 3.70 (s, 4H), 3.57 (t, *J* = 5.4 Hz, 2H), 0.88 (s, 9H), 0.05 (s, 6H). ^13^C NMR (100 MHz, DMSO-*d*_6_, δ ppm): 148.25, 140.09, 137.87, 129.95, 122.82, 118.38, 114.36, 113.84, 72.59, 70.68, 70.50, 69.86, 68.26, 62.56, 38.77, 18.22, −5.41. ESI-MS *m*/*z* 436.2564 [M + H]^+^.
*2-((2-((3-(4-acetylpiperazine-1-carbonyl)-4-(2-(2-(2-hydroxyethoxy)ethoxy)ethoxy)phenyl)amino)-5-bromopyrimidin-4-yl)amino)-5-(1,1-dioxidothiomorpholino)-N-methylbenzamide* (**5b**). The method described for **5a** was used to obtain **5b** (white solid, yield 57.7%). ^1^H NMR (600 MHz, DMSO-*d*_6_) δ 10.93 (d, *J* = 8.4 Hz, 1H), 9.33 (d, *J* = 6.6 Hz, 1H), 8.79–8.69 (m, 1H), 8.42 (s, 1H), 8.21 (d, *J* = 2.7 Hz, 1H), 7.61 (dd, *J* = 9.0, 2.7 Hz, 1H), 7.55–7.47 (m, 1H), 7.25 (d, *J* = 2.9 Hz, 1H), 7.23–7.15 (m, 1H), 7.02 (dd, *J* = 9.0, 5.7 Hz, 1H), 4.57 (t, *J* = 5.5 Hz, 1H), 4.09 (s, 2H), 3.83 (t, *J* = 5.2 Hz, 4H), 3.72 (q, *J* = 5.6 Hz, 2H), 3.56 (t, *J* = 5.3 Hz, 2H), 3.49 (dt, *J* = 12.8, 5.1 Hz, 8H), 3.43–3.37 (m, 6H), 3.20 (d, *J* = 5.9 Hz, 4H), 2.80 (d, *J* = 4.5 Hz, 3H), 2.00 (d, *J* = 40.3 Hz, 3H). ^13^C NMR (151 MHz, DMSO-*d*_6_) δ 169.30, 168.89, 167.05, 158.71, 156.14, 149.50, 143.00, 131.78, 126.10, 123.49, 123.16, 119.76, 119.25, 119.13, 115.91, 115.58, 113.25, 93.88, 72.89, 70.48, 70.28, 70.23, 69.74, 69.57, 68.46, 60.73, 50.35, 47.59, 26.75, 21.76. ESI-MS *m*/*z* 833.1687 [M + H]^+^.
*2-(2-(2-(2-(4-acetylpiperazine-1-carbonyl)-4-((5-bromo-4-((4-(1,1-dioxidothiomorpholino)-2-(methylcarbamoyl)phenyl)amino)pyrimidin-2-yl)amino)phenoxy)ethoxy)ethoxy)ethyl 4-methylbenzenesulfonate* (**6b**). The method described for **6a** was used to obtain **6b**. (white solid, yield 74.5%). ^1^H NMR (600 MHz, DMSO-*d*_6_) δ 10.90 (d, *J* = 8.5 Hz, 1H), 9.30 (d, *J* = 7.1 Hz, 1H), 8.68 (s, 1H), 8.39 (s, 1H), 8.17 (s, 1H), 7.74 (d, *J* = 7.7 Hz, 2H), 7.57 (d, *J* = 9.7 Hz, 1H), 7.48–7.44 (m, 1H), 7.42 (d, J = 8.3 Hz, 2H), 7.22 (s, 1H), 7.17 (d, *J* = 9.0 Hz, 1H), 6.98 (t, *J* = 7.6 Hz, 1H), 4.09–3.99 (m, 4H), 3.82–3.75 (m, 4H), 3.72–3.60 (m, 4H), 3.56–3.48 (m, 4H), 3.47–3.42 (m, 4H), 3.40–3.37 (m, 2H), 3.37–3.33 (m, 2H), 3.21–3.13 (m, 4H), 2.77 (d, *J* = 4.6 Hz, 3H), 2.36 (s, 3H), 1.96 (d, 3H). ^13^C NMR (101 MHz, DMSO-*d*_6_) δ 169.30, 168.86, 158.69, 156.13, 149.61, 145.44, 142.99, 132.90, 131.77, 128.58, 126.03, 123.11, 119.71, 115.58, 94.92, 70.47, 70.31, 69.55, 68.43, 55.46, 50.32, 49.12, 47.56, 45.95, 26.75, 21.61, 9.00. ESI-MS *m*/*z* 987.2181 [M + H]^+^.
*2-((2-((3-(4-acetylpiperazine-1-carbonyl)-4-(2-(2-(2-fluoroethoxy)ethoxy)ethoxy)phenyl)amino)-5-bromopyrimidin-4-yl)amino)-5-(1,1-dioxidothiomorpholino)-N-methylbenzamide* (**7b**). The method described for **7a** was used to obtain **7b** (white solid, 71.3%). *R*_f_ (MeOH/DCM = 1/10) = 0.42. ^1^H NMR (400 MHz, DMSO-*d*_6_) δ 10.89 (d, *J* = 5.3 Hz, 1H), 9.30 (d, *J* = 4.3 Hz, 1H), 8.68 (q, *J* = 4.7 Hz, 1H), 8.38 (s, 1H), 8.17 (s, 1H), 7.57 (dd, *J* = 8.9, 2.7 Hz, 1H), 7.45 (d, *J* = 9.3 Hz, 1H), 7.22 (d, *J* = 2.9 Hz, 1H), 7.17 (d, *J* = 9.3 Hz, 1H), 6.98 (dd, *J* = 9.1, 4.1 Hz, 1H), 4.54–4.51 (m, 1H), 4.42–4.37 (m, 1H), 4.09–4.03 (m, 2H), 3.79 (t, *J* = 5.1 Hz, 4H), 3.68 (d, *J* = 4.5 Hz, 3H), 3.62 (t, *J* = 4.0 Hz, 2H), 3.58–3.52 (m, 4H), 3.52–3.46 (m, 5H), 3.38–3.36 (m, 2H), 3.17–3.13 (m, 4H), 2.77 (d, *J* = 4.4 Hz, 3H), 1.96 (d, *J* = 27.0 Hz, 3H).^13^C NMR (100 MHz, DMSO-*d*_6_, δ ppm):168.76, 168.34, 166.43, 158.15, 156.99, 155.61, 149.00, 142.49, 133.81, 131.23, 129.64, 125.52, 122.96, 122.65, 121.65, 119.21, 118.77, 118.62, 115.03, 112.70, 93.79, 83.83, 82.18, 69.89, 69.77, 69.58, 69.04, 68.00, 49.80, 47.04, 28.99, 26.21, 22.08, 21.19, 13.93. The ESI-HRMS *m*/*z* calculated for C_35_H_45_BrFN_8_O_8_S^+^ 835.2243 was found to be 835.1687. IR for **7b** (KBr, cm^−1^): 3290 (vw), 2920 (s), 2850 (m), 1630 (m), 1560 (s), 1490 (w), 1460 (m), 1410 (s), 1250 (s), 1120 (s), 1050 (m), 995 (m), 949 (w), 856 (w), 818 (w), 774 (m), 717 (w), 646 (w), 519 (m), 539 (m), 594 (w).
*2-((5-bromo-2-((4-(2-(2-(2-hydroxyethoxy)ethoxy)ethoxy)-3-(1-methyl-1H-pyrazol-4-yl)phenyl)amino)pyrimidin-4-yl)amino)-5-(1,1-dioxidothiomorpholino)-N-methylbenzamide* (**5c**). The method described for **5a** was used to obtain **5c** (white solid, 48.6%). ^1^H NMR (600 MHz, DMSO-*d*_6_) δ 10.87 (s, 1H), 9.11 (s, 1H), 8.66 (d, *J* = 5.1 Hz, 1H), 8.36 (s, 1H), 8.15 (s, 1H), 8.05 (s, 1H), 7.76 (s, 2H), 7.31–7.23 (m, 1H), 7.22–7.14 (m, 1H), 6.93 (d, *J* = 8.8 Hz, 1H), 6.66 (s, 1H), 4.57 (t, *J* = 5.6 Hz, 1H), 4.17–4.05 (m, 2H), 3.82 (s, 3H), 3.81–3.79 (m, 2H), 3.71–3.65 (m, 4H), 3.64–3.60 (m, 2H), 3.58–3.55 (m, 2H), 3.47–3.44 (m, 2H), 3.43–3.39 (m, 2H), 3.13–3.07 (m, 4H), 2.76 (d, *J* = 4.5 Hz, 3H). ^13^C NMR (100 MHz, DMSO-*d*_6_, δ ppm): 169.30, 159.20, 157.73, 156.05, 150.66, 142.82, 138.02, 133.95, 131.86, 130.32, 123.50, 123.03, 121.66, 120.05, 119.14, 118.35, 115.50, 113.38, 93.80, 72.93, 70.42, 70.32, 69.60, 68.14, 60.75, 55.45, 50.30, 47.44, 39.08, 26.75. ESI-MS *m*/*z* 731.1633 [M + H]^+^.
*2-(2-(2-(4-((5-bromo-4-((4-(1,1-dioxidothiomorpholino)-2-(methylcarbamoyl)phenyl)amino)pyrimidin-2-yl)amino)-2-(1-methyl-1H-pyrazol-4-yl)phenoxy)ethoxy)ethoxy)ethyl 4-methylbenzenesulfonate* (**6c**). The method described for **6a** was used to obtain **6c** (white solid, 72.1%). ^1^H NMR (600 MHz, DMSO-*d*_6_) δ 10.88 (s, 1H), 9.13 (s, 1H), 8.68 (d, *J* = 4.8 Hz, 1H), 8.36 (s, 1H), 8.15 (s, 1H), 8.03 (d, *J* = 5.8 Hz, 1H), 7.76 (d, *J* = 2.7 Hz, 1H), 7.75–7.69 (m, 2H), 7.56–7.49 (m, 1H), 7.40 (d, *J* = 8.1 Hz, 1H), 7.31 (d, *J* = 7.9 Hz, 1H), 7.29–7.22 (m, 1H), 7.18 (d, J = 2.5 Hz, 1H), 6.93 (dd, *J* = 8.9, 2.8 Hz, 1H), 6.64 (s, 1H), 4.11–4.04 (m, 3H), 3.83–3.75 (m, 5H), 3.74–3.69 (m, 1H), 3.68–3.62 (m, 4H), 3.62–3.47 (m, 6H), 3.09 (d, *J* = 5.9 Hz, 4H), 2.76 (d, *J* = 4.5 Hz, 3H), 2.31 (d, *J* = 32.0 Hz, 3H). ^13^C NMR (151 MHz, DMSO-*d*_6_) δ 168.60, 155.83, 151.10, 145.72, 142.87, 137.60, 137.49, 129.84, 129.43, 128.04, 127.57, 125.49, 124.47, 123.33, 122.99, 117.60, 114.71, 112.92, 93.33, 72.38, 70.53, 69.88, 69.77, 69.09, 60.20, 46.66, 43.51, 38.56, 26.22, 20.76. ESI-MS *m*/*z* 897.1796 [M + H]^+^.
*2-((5-bromo-2-((4-(2-(2-(2-fluoroethoxy)ethoxy)ethoxy)-3-(1-methyl-1H-pyrazol-4-yl)phenyl)amino)pyrimidin-4-yl)amino)-5-(1,1-dioxidothiomorpholino)-N-methylbenzamide* (**7c**). The method described for **7a** was used to obtain **7c** (white solid, 68.2%). *R*_f_ (MeOH/DCM = 1/10) = 0.53. ^1^H NMR (400 MHz, DMSO-*d*_6_) δ 10.91 (s, 1H), 9.14 (s, 1H), 8.78–8.68 (m, 1H), 8.46–8.35 (m, 1H), 8.19 (s, 1H), 8.09 (s, 1H), 7.85–7.69 (m, 2H), 7.34–7.26 (m, 1H), 7.27–7.20 (m, 1H), 6.97 (d, *J* = 8.9 Hz, 1H), 6.70 (s, 1H), 4.57 (t, *J* = 4.0 Hz, 1H), 4.45 (t, *J* = 4.0 Hz, 1H), 4.19–4.08 (m, 2H), 3.85 (s, 5H), 3.74–3.69 (m, 4H), 3.69–3.61 (m, 6H), 3.16–3.08 (m, 4H), 2.80 (d, *J* = 4.4 Hz, 3H). ^13^C NMR (100 MHz, DMSO-*d*_6_, δ ppm): 169.30, 159.21, 157.73, 156.03, 150.06, 142.82, 137.96, 133.94, 131.87, 130.31, 123.50, 123.02, 121.66, 120.35, 120.05, 119.13, 118.35, 115.51, 113.36, 93.79, 84.40, 82.75, 70.47, 70.35, 70.14, 69.69, 68.13, 50.31, 47.44, 39.06, 26.74. The ESI-HRMS *m*/*z* calculated for C_32_H_39_BrFN_8_O_6_S^+^ 761.1875 was found to be 761.1875. IR for **7c** (KBr, cm^−1^): 3260 (vw), 2920 (vw), 1260 (w), 1520 (s), 1420 (m), 1270 (m), 1120 (s), 1050 (w), 1000 (w), 949 (w), 870 (m), 810 (m), 775 (m), 714 (m), 668 (m), 536 (m).
*2-((5-bromo-2-((4-(2-(2-(2-hydroxyethoxy)ethoxy)ethoxy)-3-(1-methyl-1H-pyrazol-4-yl)phenyl)amino)pyrimidin-4-yl)amino)-5-(4-ethylpiperazin-1-yl)-N-methylbenzamide* (**5g**). The method described for **5a** was used to obtain **5g**. (white solid, yield 73.5%). ^1^H NMR (400 MHz, DMSO-*d*_6_) δ 9.21 (s, 1H), 9.04 (s, 1H), 8.59 (s, 1H), 8.13 (s, 1H), 8.00 (s, 1H), 7.78–7.70 (m, 2H), 7.67 (d, *J* = 2.6 Hz, 1H), 7.57 (s, 1H), 7.26 (d, *J* = 8.9 Hz, 1H), 6.88 (d, *J* = 8.7 Hz, 1H), 6.81 (d, *J* = 8.9 Hz, 1H), 4.59 (s, 1H), 4.07–4.03 (m, 2H), 3.82 (s, 3H), 3.80–3.76 (m, 2H), 3.63–3.58 (m, 2H), 3.57–3.52 (m, 2H), 3.49–3.42 (m, 2H), 3.42–3.37 (m, 2H), 3.28–3.25 (m, 2H), 3.13 (d, *J* = 5.1 Hz, 2H), 2.79 (d, *J* = 4.7 Hz, 2H), 2.78–2.73 (m, 3H), 2.58–2.48 (m, 2H), 2.43–2.31 (m, 2H), 1.01 (t, *J* = 7.1 Hz, 3H). ^13^C NMR (101 MHz, DMSO-*d*_6_) δ 167.04, 158.95, 158.16, 157.09, 150.24, 137.79, 134.94, 134.11, 130.29, 126.72, 125.32, 121.54, 120.31, 119.28, 118.31, 113.30, 92.60, 72.94, 70.42, 69.65, 68.06, 60.74, 53.10, 52.03, 39.09, 26.37, 12.50. The ESI-MS *m*/*z* calculated for C_34_H_45_BrN_9_O_5_^+^ 738.2722 was found to be 738.2655.
*2-(2-(2-(4-((5-bromo-4-((4-(4-ethylpiperazin-1-yl)-2-(methylcarbamoyl)phenyl)amino)pyrimidin-2-yl)amino)-2-(1-methyl-1H-pyrazol-4-yl)phenoxy)ethoxy)ethoxy)ethyl 4-methylbenzenesulfonate* (**6g**). The method described for **6a** was used to obtain **6g** (white solid, yield 69.8%). ^1^H NMR (600 MHz, DMSO-*d*_6_) δ 9.39–9.19 (m, 1H), 9.09 (s, 1H), 8.64 (s, 1H), 8.17 (s, 1H), 8.02 (s, 1H), 7.84–7.77 (m, 2H), 7.76–7.74 (m, 2H), 7.73–7.70 (m, 1H), 7.60 (s, 1H), 7.45–7.41 (m, 2H), 7.33 (s, 1H), 6.92 (s, 1H), 6.85 (d, *J* = 8.9 Hz, 1H), 4.13–4.05 (m, 4H), 3.84 (s, 3H), 3.79 (t, *J* = 4.7 Hz, 2H), 3.62–3.56 (m, 4H), 3.54–3.49 (m, 2H), 3.37–3.34 (m, 2H), 3.33–3.30 (m, 2H), 3.20–3.09 (m, 2H), 2.87–2.75 (m, 5H), 2.55–2.51 (m, 1H), 2.49–2.46 (m, 1H), 2.37 (s, 3H), 1.26–1.17 (m, 3H). ^13^C NMR (101 MHz, DMSO-*d*_6_) δ 167.64, 166.96, 158.96, 158.14, 157.09, 150.22, 145.42, 139.19, 137.74, 134.93, 134.12, 132.90, 130.65, 130.23, 128.12, 127.24, 126.76, 126.62, 126.12, 125.35, 121.54, 120.37, 119.27, 118.30, 118.07, 113.28, 70.44, 70.36, 70.21, 69.65, 68.46, 68.03, 55.47, 53.29, 52.93, 52.44, 52.07, 50.76, 26.32, 21.58, 12.53. ESI-MS *m*/*z* 892.2792 [M + H]^+^.
*2-((5-bromo-2-((4-(2-(2-(2-fluoroethoxy)ethoxy)ethoxy)-3-(1-methyl-1H-pyrazol-4-yl)phenyl)amino)pyrimidin-4-yl)amino)-5-(4-ethylpiperazin-1-yl)-N-methylbenzamide* (**7g**). The method described for **7a** was used to obtain **7g** (white solid, yield 57.6%). *R*_f_ (MeOH/DCM = 1/5) = 0.66. ^1^H NMR (600 MHz, DMSO-*d*_6_) δ 9.27 (d, *J* = 4.7 Hz, 1H), 9.09 (s, 1H), 8.63 (s, 1H), 8.17 (s, 1H), 8.04 (d, *J* = 2.6 Hz, 1H), 7.81 (d, *J* = 2.8 Hz, 1H), 7.78 (d, *J* = 8.8 Hz, 1H), 7.71 (d, *J* = 2.7 Hz, 1H), 7.60 (s, 1H), 7.31 (s, 1H), 6.93 (s, 1H), 6.85 (d, *J* = 9.1 Hz, 1H), 4.09 (dd, *J* = 5.8, 3.5 Hz, 2H), 3.86 (d, *J* = 3.0 Hz, 3H), 3.85–3.81 (m, 2H), 3.69 (qd, *J* = 4.1, 1.8 Hz, 3H), 3.66 (dd, *J* = 6.1, 3.7 Hz, 2H), 3.64–3.61 (m, 2H), 3.21–3.12 (m, 3H), 2.83 (d, *J* = 4.8 Hz, 3H), 2.80 (d, *J* = 5.2 Hz, 4H), 2.52 (s, 2H), 2.40 (q, *J* = 7.2 Hz, 2H), 1.04 (t, *J* = 7.2 Hz, 3H). ^13^C NMR (151 MHz, DMSO-*D*_6_) δ 166.96, 158.97, 158.11, 157.09, 150.24, 147.11, 137.75, 134.92, 134.11, 130.24, 128.98, 126.75, 125.35, 121.55, 120.39, 119.30, 118.31, 113.29, 84.11, 83.01, 71.10, 70.48, 70.33, 70.30, 70.18, 69.67, 58.07, 55.46, 53.33, 52.96, 52.10, 44.07, 39.10, 39.06, 23.60, 14.02. The ESI-HRMS *m*/*z* calculated for C34H44BrFN9O4^+^ 740.2678 was found to be 740.2682. IR for **7g** (KBr, cm^−1^): 3400 (m), 2920 (s), 2850 (m), 1650 (s), 1610 (m), 1530 (s), 1490 (s), 1410 (s), 1290 (m), 1110 (s), 1050 (w), 942 (m), 838 (m), 813 (m), 773 (m), 691 (m), 666 (m), 625 (m), 555 (m).
*5-(4-acetylpiperazin-1-yl)-2-((5-bromo-2-((4-(2-(2-(2-hydroxyethoxy)ethoxy)ethoxy)-3-(1-methyl-1H-pyrazol-4-yl)phenyl)amino)pyrimidin-4-yl)amino)-N-methylbenzamide* (**5e**). The method described for **5a** was used to obtain **5e** (white solid, 66.8%). ^1^H NMR (600 MHz, DMSO-*d*_6_) δ 9.09 (s, 1H), 9.02 (d, *J* = 4.7 Hz, 1H), 8.63 (s, 1H), 8.17 (s, 1H), 8.05 (s, 1H), 7.78 (d, *J* = 2.3 Hz, 2H), 7.71 (d, *J* = 2.8 Hz, 1H), 7.59 (s, 1H), 7.35–7.18 (m, 1H), 6.90 (s, 1H), 6.85 (d, *J* = 8.9 Hz, 1H), 4.61 (t, *J* = 5.5 Hz, 1H), 4.10–4.06 (m, 2H), 3.85 (s, 3H), 3.83–3.80 (m, 2H), 3.67–3.63 (m, 2H), 3.62–3.57 (m, 6H), 3.50–3.47 (m, 2H), 3.45–3.42 (m, 2H), 2.85–2.82 (m, 3H), 2.82–2.77 (m, 2H), 2.75–2.70 (m, 2H), 2.05 (s, 3H). ^13^C NMR (100 MHz, DMSO-*d*_6_, δ ppm): 168.43, 166.61, 158.42, 157.64, 156.56, 149.70, 146.14, 137.24, 134.48, 133.60, 129.77, 128.97, 126.13, 124.78, 121.00, 119.81, 118.72, 118.66, 117.79, 112.79, 72.40, 69.89, 69.13, 67.56, 60.22, 52.74, 52.07, 45.93, 41.08, 38.55, 25.92, 21.26. ESI-MS *m*/*z* 752.2333 [M + H]^+^.
*2-(2-(2-(4-((4-((4-(4-acetylpiperazin-1-yl)-2-(methylcarbamoyl)phenyl)amino)-5-bromopyrimidin-2-yl)amino)-2-(1-methyl-1H-pyrazol-4-yl)phenoxy)ethoxy)ethoxy)ethyl 4-methylbenzenesulfonate* (**6e**). The method described for **6a** was used to obtain **6e** (white solid, 81.2%). ^1^H NMR (600 MHz, DMSO-*d*_6_) δ 9.10 (s, 1H), 9.02 (d, *J* = 4.9 Hz, 1H), 8.65 (s, 1H), 8.17 (s, 1H), 8.01 (s, 1H), 7.81–7.76 (m, 2H), 7.75–7.73 (m, 2H), 7.71 (d, *J* = 2.7 Hz, 1H), 7.60–7.54 (m, 1H), 7.44–7.39 (m, 2H), 7.34–7.27 (m, 1H), 6.88 (s, 1H), 6.84 (d, *J* = 8.9 Hz, 1H), 4.10–4.06 (m, 4H), 3.82 (s, 3H), 3.80–3.77 (m, 2H), 3.61–3.57 (m, 8H), 3.52–3.49 (m, 2H), 2.83 (d, *J* = 4.7 Hz, 3H), 2.79 (s, 2H), 2.72 (s, 2H), 2.36 (s, 3H), 2.03 (s, 3H). ^13^C NMR (151 MHz, DMSO-*d*_6_) δ 168.22, 166.43, 158.26, 157.42, 156.39, 149.54, 145.96, 144.69, 137.05, 134.34, 133.46, 132.24, 129.92, 129.54, 128.81, 127.40, 125.92, 124.62, 120.87, 119.64, 118.54, 118.49, 117.64, 112.63, 91.99, 69.73, 69.67, 69.53, 68.97, 67.77, 67.38, 54.74, 52.56, 51.90, 45.77, 40.92, 38.34, 25.72, 25.62, 21.04, 20.86. ESI-MS *m*/*z* 906.2981 [M + H]^+^.
*5-(4-acetylpiperazin-1-yl)-2-((5-bromo-2-((4-(2-(2-(2-fluoroethoxy)ethoxy)ethoxy)-3-(1-methyl-1H-pyrazol-4-yl)phenyl)amino)pyrimidin-4-yl)amino)-N-methylbenzamide* (**7e**). *R*_f_ (MeOH/DCM = 1/10) = 0.51. The method described for **7a** was used to obtain **7e** (white solid, 58.1%). ^1^H NMR (400 MHz, DMSO-*d*_6_) δ 9.06 (s, 1H), 8.98 (q, *J* = 4.8 Hz, 1H), 8.60 (s, 1H), 8.13 (s, 1H), 8.00 (s, 1H), 7.81–7.71 (m, 2H), 7.67 (d, *J* = 2.7 Hz, 1H), 7.54 (s, 1H), 7.34–7.21 (m, 1H), 6.89–6.83 (m, 1H), 6.81 (d, *J* = 8.9 Hz, 1H), 4.57–4.48 (m, 1H), 4.43–4.35 (m, 1H), 4.12–4.00 (m, 2H), 3.89–3.74 (m, 5H), 3.68–3.64 (m, 1H), 3.64–3.60 (m, 2H), 3.60–3.52 (m, 7H), 2.80 (d, *J* = 4.7 Hz, 3H), 2.76 (t, *J* = 5.0 Hz, 2H), 2.69 (t, *J* = 5.1 Hz, 2H), 2.01 (s, 3H). ^13^C NMR (100 MHz, DMSO-*d*_6_, δ ppm): 168.39, 116.59, 158.30, 157.38, 156.56, 149.74, 146.13, 137.18, 134.43, 133.50, 129.73, 128.96, 126.10, 124.77, 121.01, 119.80, 118.74, 117.76, 112.78, 92.12, 83.84, 82.19, 69.93, 69.79, 69.59, 69.12, 67.55, 52.72, 52.03, 45.91, 41.06, 38.50, 25.88, 21.20. The ESI-HRMS *m*/*z* calculated for C_34_H_42_BrFN_9_O_5_^+^ 754.2476 was found to be 754.2477. IR for **7e** (KBr, cm^−1^): 3310 (w), 2870 (w), 1630 (m), 1560 (m), 1520 (s), 1410 (s), 1220 (m), 1130 (m), 1070 (m), 984 (m), 939 (w), 891 (w), 852 (w), 773 (w), 732 (w), 672 (w), 611 (w), 590 (w), 552 (w), 520 (w).

### 4.2. Radiolabeling

Radiopharmaceutical purification was performed on a Shimadzu (Tokyo, Japan) LC-20AT high-performance liquid chromatograph equipped with a Bioscan (Amman, Jordan) Flow count 3200 NaI/PMT γ- radiation scintillation detector.

#### 4.2.1. Drying Produce for [^18^F]-Fluoride

To acquire an aqueous [^18^F] fluoride solution, a solution containing kryptofix 2.2.2. (10 mg) and potassium carbonate (5 mg) in 0.4 mL of acetonitrile was added, and the mixture was dried in a stream of argon at 100 °C for 10 min, followed by azeotrope with acetonitrile (3 × 0.5 mL) three times.

#### 4.2.2. Synthesis of [^18^F]**7a** and [^18^F]**7c**

The solution of precursor (**6a** or **6c**, 2 mg) in acetonitrile (0.5 mL) was added to the above in a sealed vial containing the dried [^18^F]-fluoride, and it was heated at 100 °C for 20 min. The mixture was diluted with water (10 mL) and passed through a Sep-Pak C18 cartridge. Then, the column was washed with additional water (10 mL), the residual crude product was eluted with acetonitrile (2 mL), and the purification of the solution was accomplished using HPLC (acetonitrile/H_2_O = 1.65: 0.35, flow rate: 2 mL/min). The preparation time was around 50 min, and the overall radiochemical yields were 15.43% and 17.28%, respectively. The radioactive product was purified using a Shimadzu LC-20AT HPLC apparatus equipped with an SPD-20A UV detector (λ = 254 nm) and a Bioscan flow count 3200 NaI/PMT γ-radiation scintillation detector. A C18 reverse-phase semipreparative column from H&E CORPORATION was employed for chromatographic separation, with specifications of a 5 μm particle size, a 100 Å pore size, and dimensions of 10 × 250 mm.

### 4.3. FAK Inhibitory Assay and Partition Coefficient Determination

The FAK kinase inhibition test and the partition coefficient test were conducted according to a previously reported method [10].

### 4.4. Molecular Docking

We optimized our small-molecule ligands and proteins using Sybyl X V2.0 software and conducted a molecular docking study using the Surflex-Dock GeomX module.

### 4.5. In Vitro Stability

The in vitro stabilities of [^18^F]**7a** and [^18^F]**7c** in saline and mouse serum were tested by measuring the radiochemistry purity using HPLC (H&E CORPORATION, Baton Rouge, LA, USA; C18, 5 μm, 100 Å, 10 × 250 mm, MeOH/H_2_O = 1.65:0.35, flow rate of 2 mL/min) after incubation at 37 °C.

### 4.6. Biodistribution Studies

Kunming mice (female, 18−22 g) and S180 ascites mice were purchased from Beijing Xinglong Animal Technology Co., Ltd., Beijing, China and Beijing Vital River Animal Technology Co., Ltd., Beijing, China, respectively. All the mice-concerned experiments were conducted in strict accordance with the “Guidelines for Humane Treatment of Laboratory Animals”, and the whole process was approved by the Animal Protection Committee of Beijing Normal University (Approval Code: BNUCC-EAW-2023-16; Approval date: 14 June 2023). The specific experimental operations were in accordance with those methods described previously [10].

## Figures and Tables

**Table 1:** In vitro enzyme activity of compound **7** and reference compound VS-6063.

Compound ID	Structure	IC_50_ (nmol)
**7a**	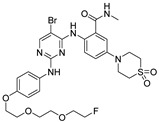	5.59
**7d**	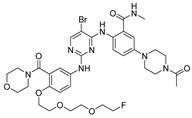	1968
**7f**	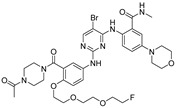	10.02
**7b**	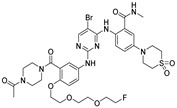	24.6
**7c**	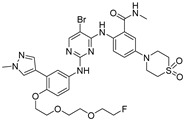	1.27
**7g**	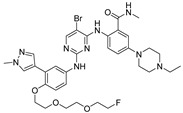	1238
**7e**	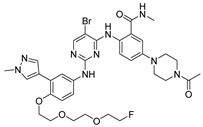	643
VS-6063	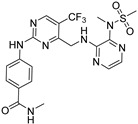	1.26

**Table 2:** Biodistributions of [^18^F]**7a** and [^18^F]**7c** in mice bearing S-180 tumors after intravenous injection *.

Tissue	[^18^F]7a			[^18^F]7c		
	30 min	60 min	120 min	30 min	60 min	120 min
Blood	0.98 ± 0.26	0.96 ± 0.10	0.92 ± 0.15	2.74 ± 0.11	3.43 ± 0.04	3.79 ± 0.46
Brine	0.57 ± 0.08	1.08 ± 0.13	0.16 ± 0.01	2.81 ± 0.06	3.12 ± 0.66	2.01 ± 0.18
Heart	3.05 ± 0.29	1.12 ± 0.03	0.85 ± 0.09	3.46 ± 0.65	2.53 ± 0.56	3.12 ± 0.25
Liver	23.49 ± 4.29	23.16 ± 2.65	8.86 ± 3.14	17.64 ± 4.45	19.21 ± 3.81	28.36 ± 5.74
Lung	33.55 ± 5.59	28.77 ± 5.72	11.63 ± 1.25	120.47 ± 15.08	128.13 ± 6.74	125.29 ± 16.25
Kidney	3.02 ± 1.70	1.47 ± 0.18	1.16 ± 0.06	4.00 ± 0.25	3.82 ± 0.44	3.50 ± 0.37
Spleen	5.43 ± 1.12	2.53 ± 0.21	5.58 ± 1.06	16.32 ± 1.26	8.48 ± 0.10	24.70 ± 4.31
Stomach	0.92 ± 0.28	0.80 ± 0.21	1.16 ± 0.40	5.81 ± 1.02	1.14 ± 0.16	3.32 ± 0.31
Bone	2.32 ± 0.45	2.96 ± 0.43	1.27 ± 0.42	4.12 ± 0.39	4.86 ± 0.30	10.70 ± 1.01
Muscle	1.63 ± 0.34	2.50 ± 0.59	0.20 ± 0.03	3.50 ± 0.18	2.80 ± 0.49	2.04 ± 0.12
Small intestine	2.95 ± 0.17	1.58 ± 0.35	0.96 ± 0.29	13.02 ± 1.93	5.73 ± 0.82	7.49 ± 1.46
Large intestine	1.46 ± 0.13	1.36 ± 0.36	4.5 ± 1.91	6.18 ± 0.60	4.47 ± 0.44	48.07 ± 9.85
Tumor	1.31 ± 0.07	1.39 ± 0.30	0.86 ± 0.34	6.58 ± 0.46	5.85 ± 0.28	5.18 ± 0.68
Tumor/blood	1.34	1.45	0.89	2.40	1.71	1.37
Tumor/muscle	0.80	0.56	4.30	1.88	2.09	2.16

* Data are expressed as the percentage of injected dose per gram (% ID/g), mean ± SD, *n* = 3.

## Data Availability

Date are contained within the article and Supplementary Materials.

## Supplementary Materials

The following supporting information can be downloaded at: https://www.mdpi.com/article/10.3390/molecules29061224/s1, Figures S1–S21: NMR and Ms spectra of **7a**–**7g**.
