# Supplementary material for: Design, Synthesis, Biological Evaluation and Molecular Docking of Novel F-18-Labeled Focal Adhesion Kinase Inhibitors as Potential Tumor Radiotracers
Source: Molecules. 2024 Mar 8;29(6):1224. doi: 10.3390/molecules29061224 (PMC10974507; doi:10.3390/molecules29061224)
Supplement: Supplementary file 1 [file molecules-29-01224-s001.zip › molecules-2894514-supplementary.pdf]

Design, Synthesis, Biological Evaluation and Molecular Docking of Novel F-18 labeled Focal Adhesion Kinase Inhibitors as Potential Tumor Radiotracers

Hailong Yang, Ye Li, Huaju Liang, Chun Cui, Lu Gan and Huabei Zhang *

Key Laboratory of Radiopharmaceuticals of Ministry of Education, College of Chemistry, Beijing Normal University, No. 19 Xinjiekouwai Street, Haidian District, Beijing 100875, China.

* Correspondence: hbzhang@bnu.edu.cn.

Supporting information

NMR and Ms spectra of **7a-7g** S1-S21

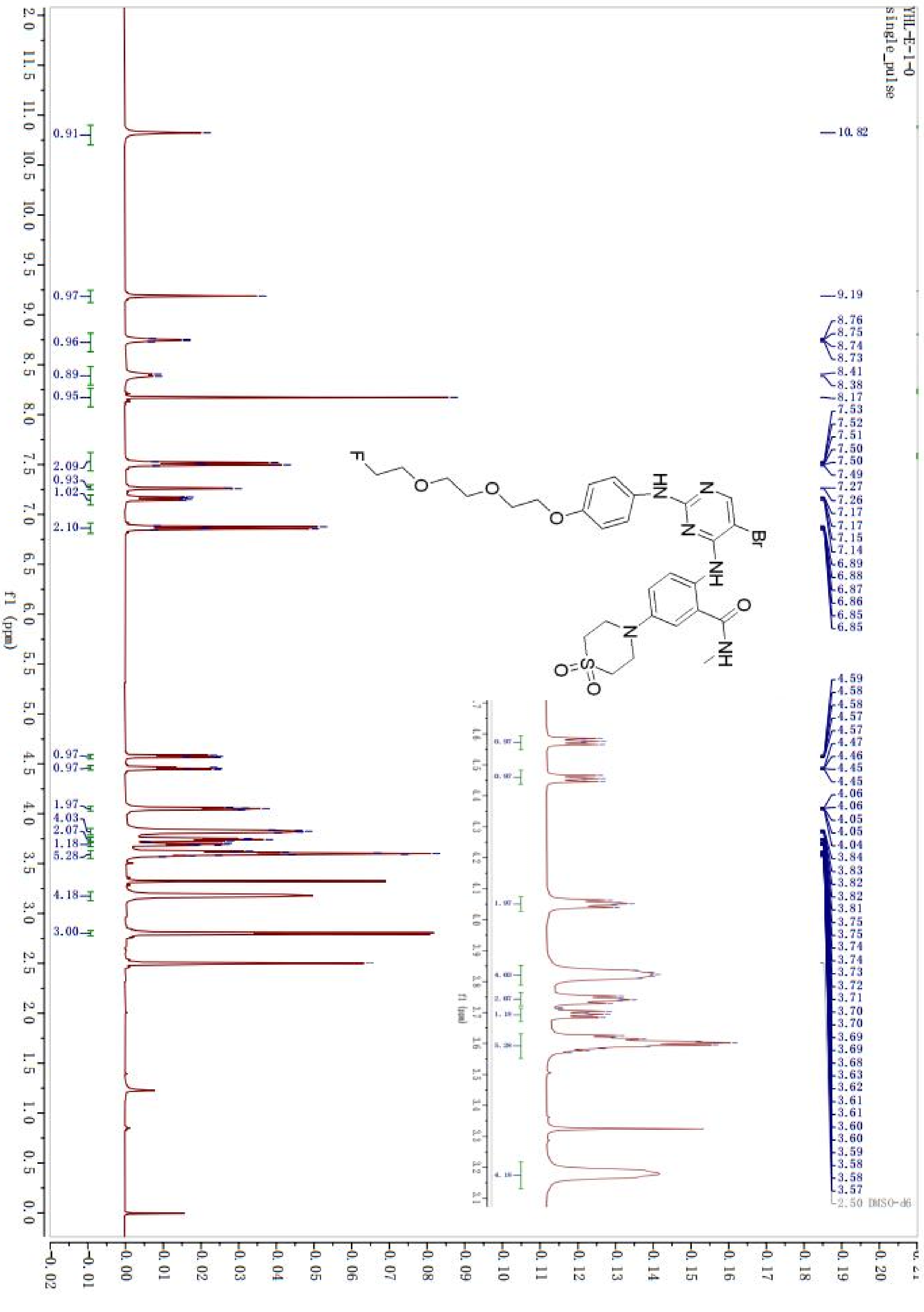

Figure S1 ^{1}H NMR spectrum of **7a**

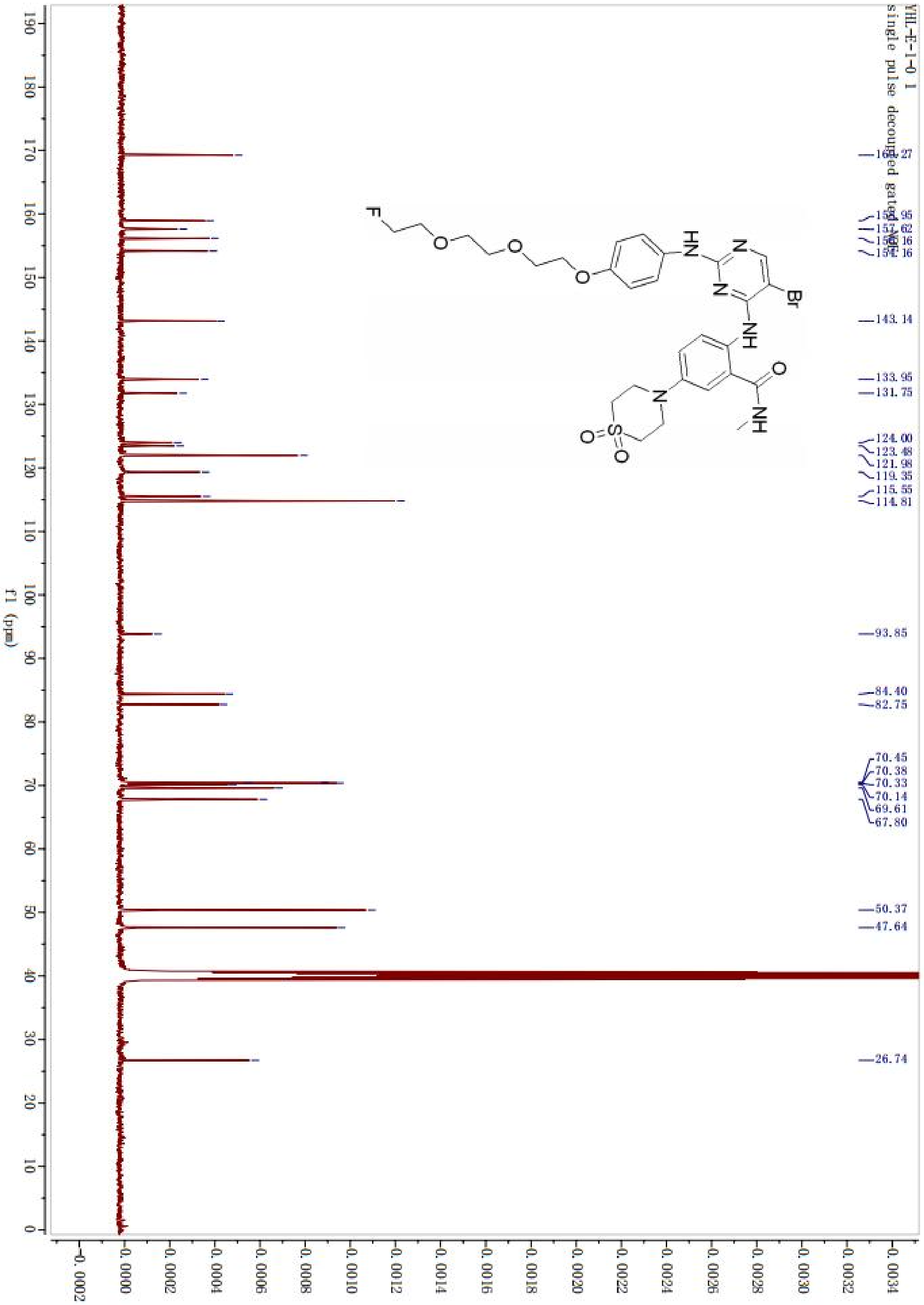

Figure S2 ^{13}C NMR spectrum of **7a**

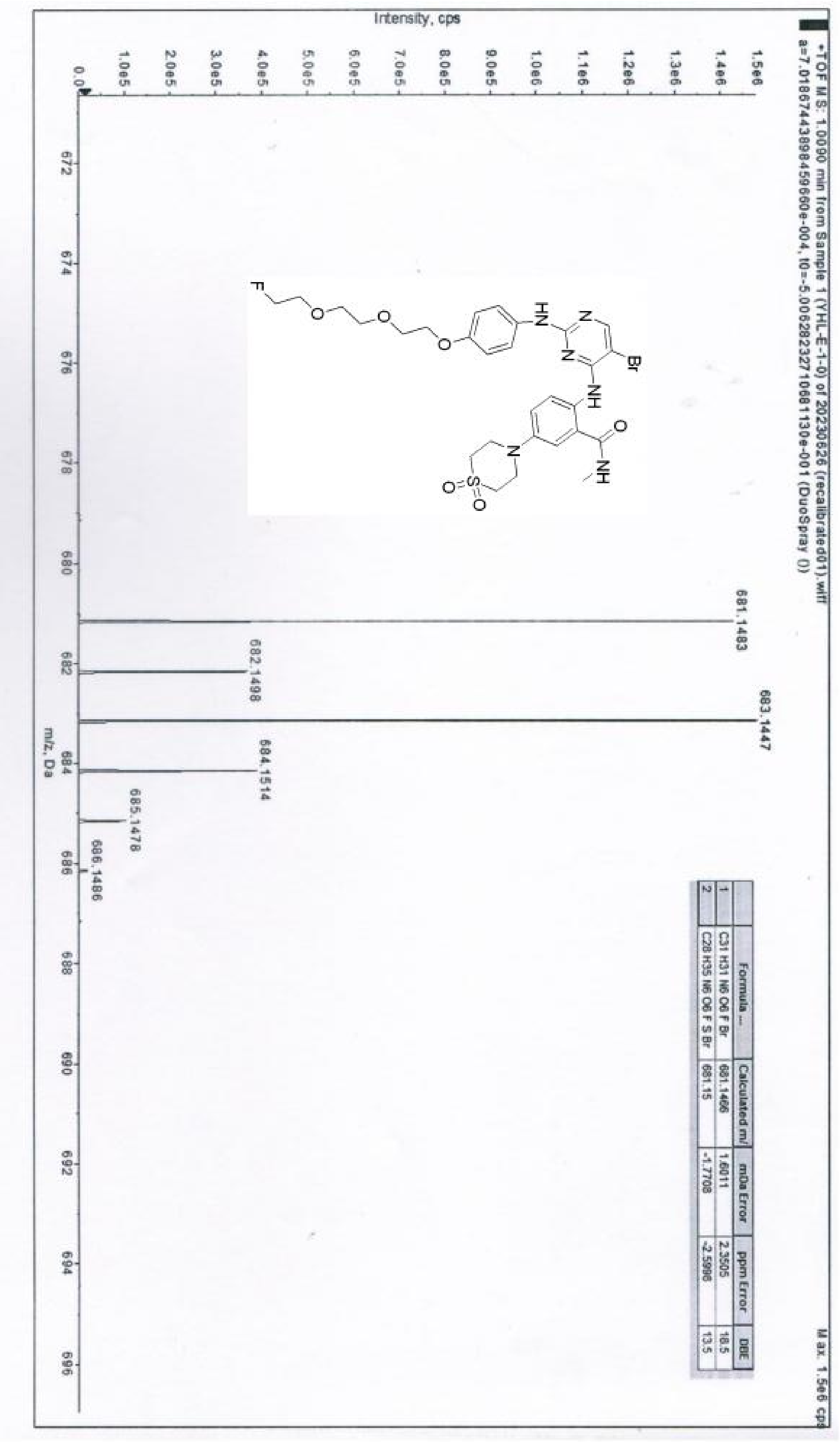

Figure S3 ESI-HRMS spectrum of **7a**

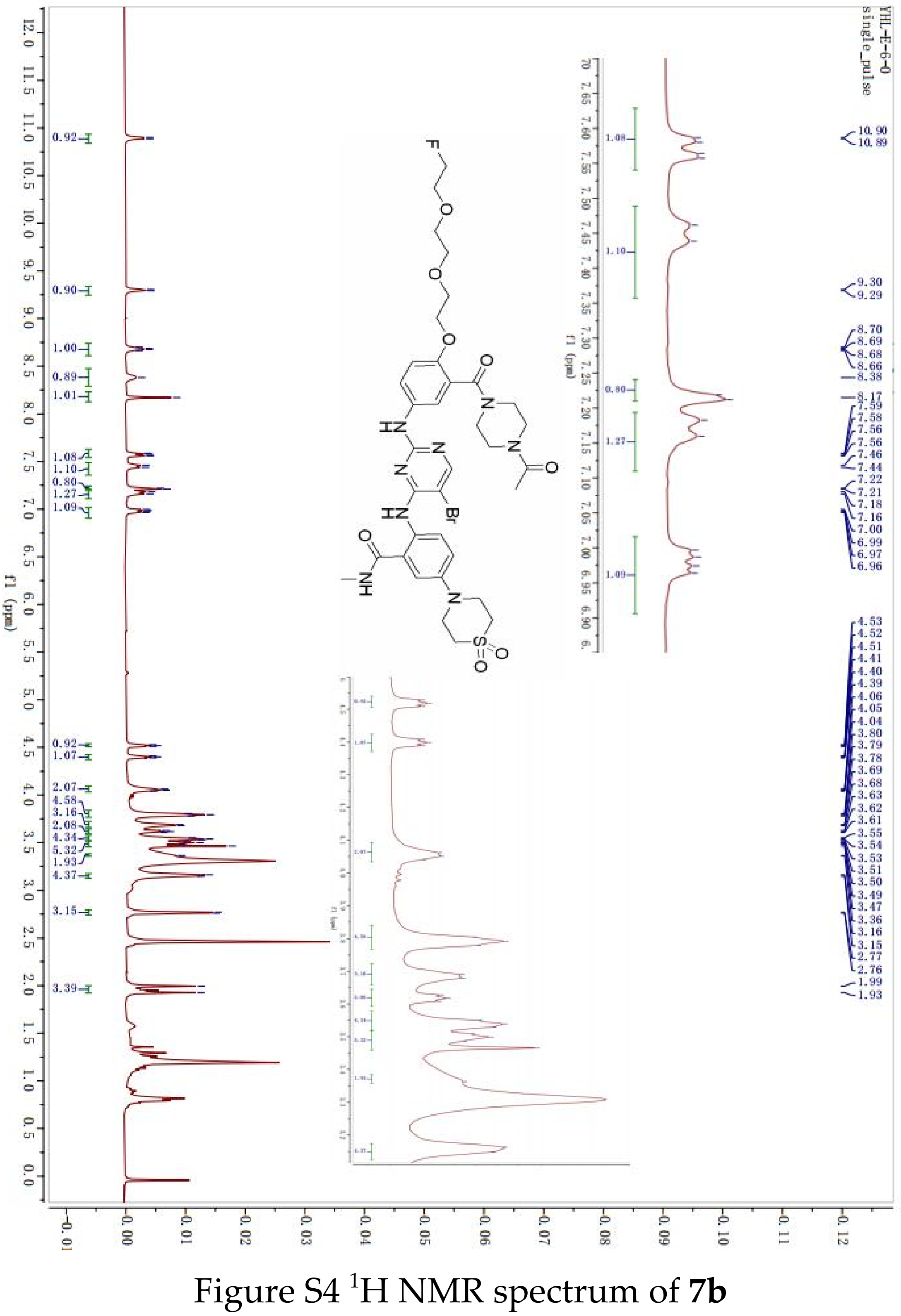

Figure S4 ^{1}H NMR spectrum of **7b**

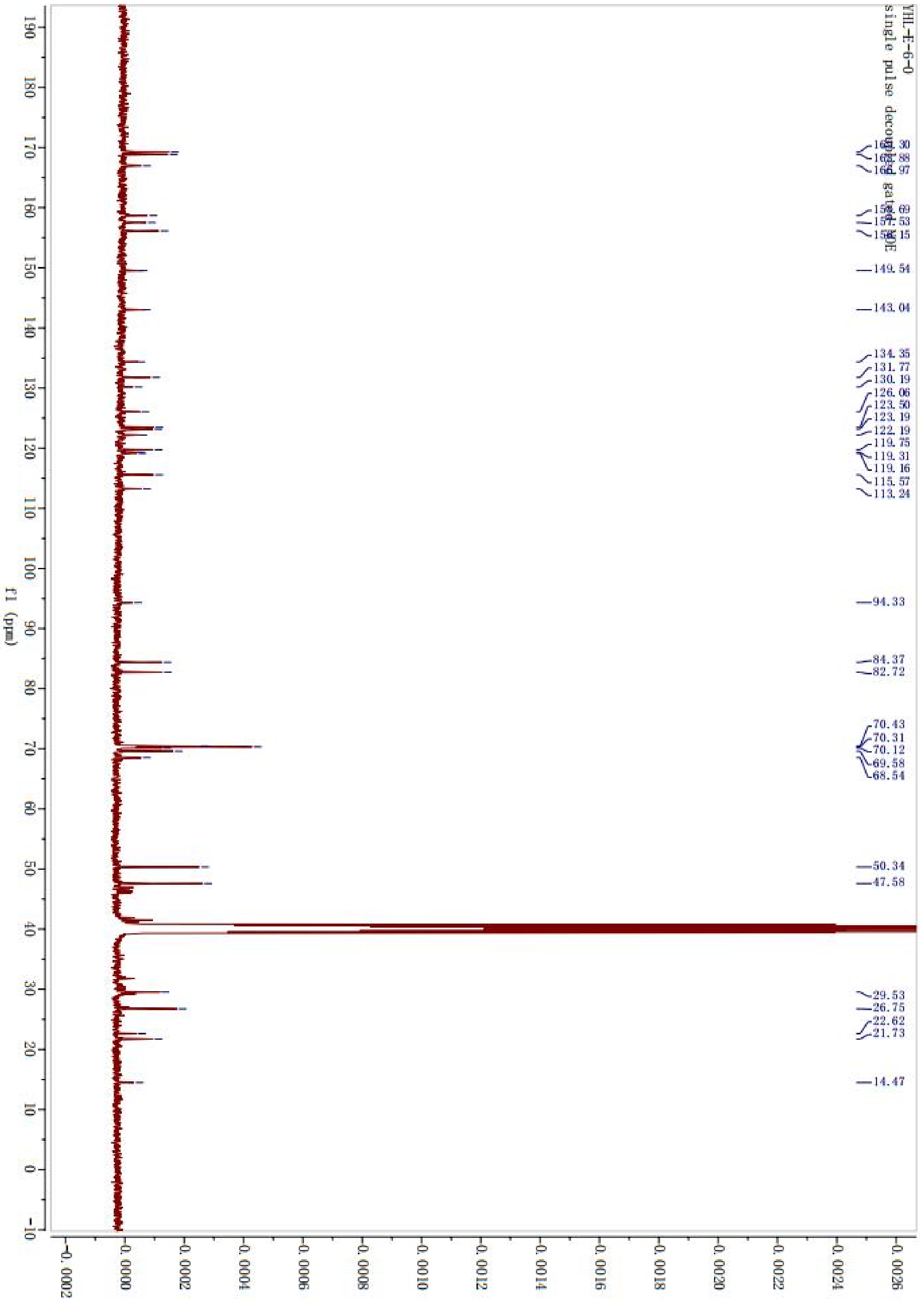

Figure S5 ^{13}C NMR spectrum of **7b**

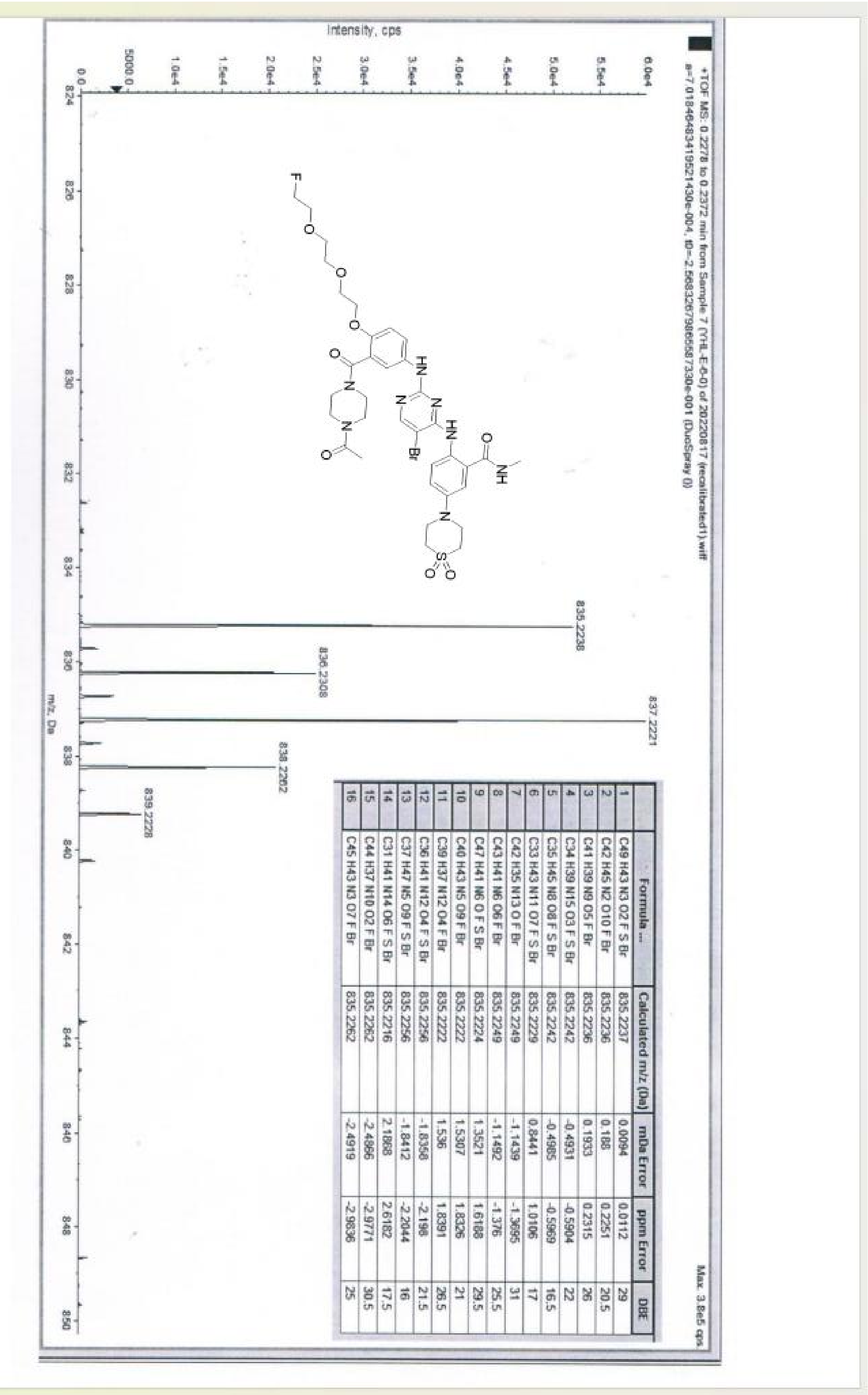

	Formula ...	Calculated m/z (Da)	mDa Error	ppm Error	DBE
1	C49 H43 N3 O2 F S Br	835.2237	0.0094	0.0112	29
2	C42 H45 N2 O10 F Br	835.2236	0.188	0.2251	20.5
3	C41 H39 N9 O5 F Br	835.2236	0.1933	0.2315	26
4	C34 H39 N15 O3 F S Br	835.2242	-0.4931	-0.5904	22
5	C35 H45 N8 O8 F S Br	835.2242	-0.4985	-0.5969	16.5
6	C33 H43 N11 O7 F S Br	835.2229	0.8441	1.0106	17
7	C42 H35 N13 O F Br	835.2249	-1.1439	-1.3695	31
8	C43 H41 N6 O6 F Br	835.2249	-1.1492	-1.376	25.5
9	C47 H41 N6 O F S Br	835.2224	1.3521	1.6188	29.5
10	C40 H43 N5 O9 F Br	835.2222	1.5307	1.8326	21
11	C39 H37 N12 O4 F Br	835.2222	1.536	1.8391	26.5
12	C36 H41 N12 O4 F S Br	835.2256	-1.8358	-2.198	21.5
13	C37 H47 N5 O9 F S Br	835.2256	-1.8412	-2.2044	16
14	C31 H41 N14 O6 F S Br	835.2216	2.1868	2.6182	17.5
15	C44 H37 N10 O2 F Br	835.2262	-2.4866	-2.9771	30.5
16	C45 H43 N3 O7 F Br	835.2262	-2.4919	-2.9836	25

Figure S6 ESI-HRMS spectrum of **7b**

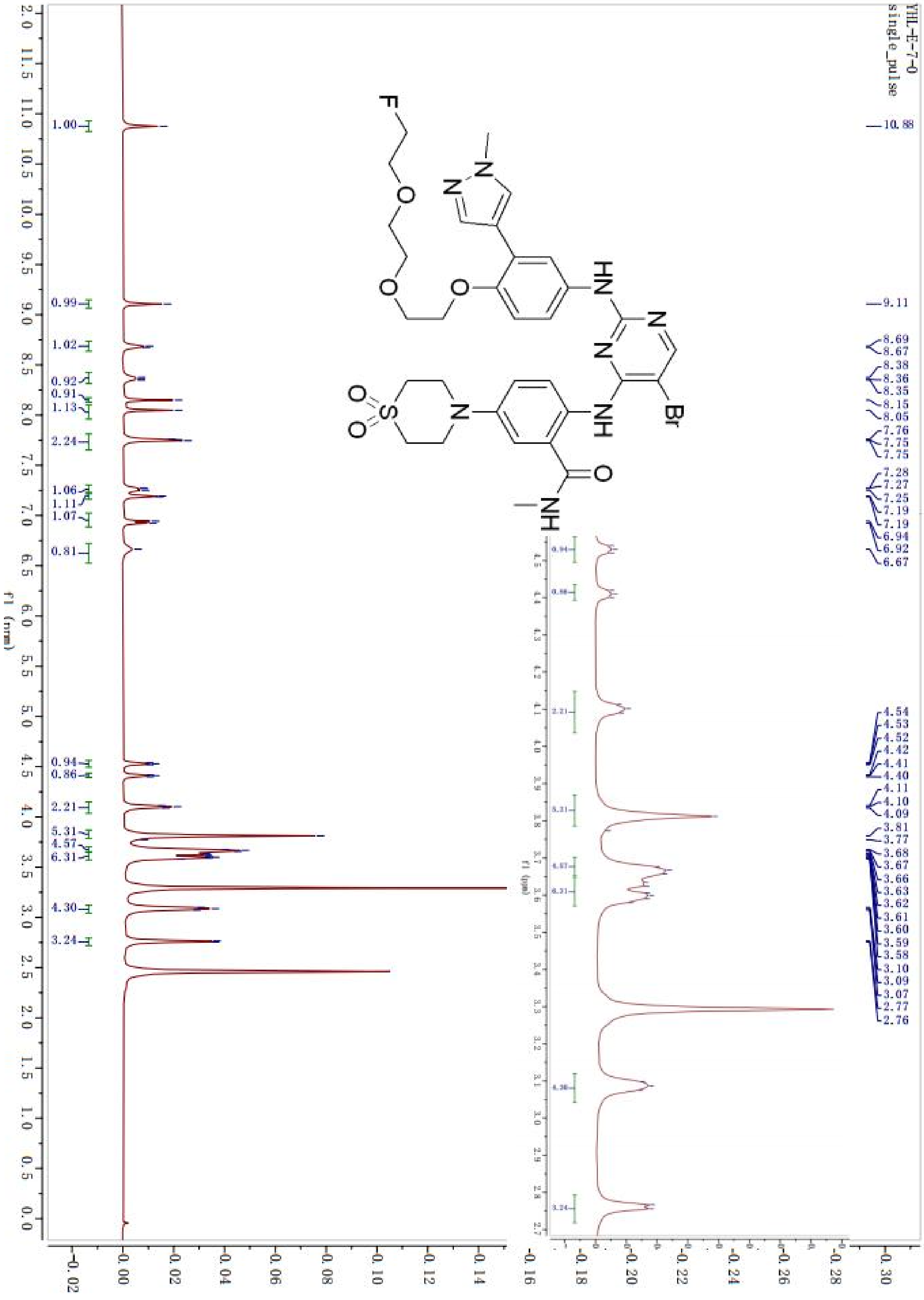

Figure S7 ^{1}H NMR spectrum of **7c**

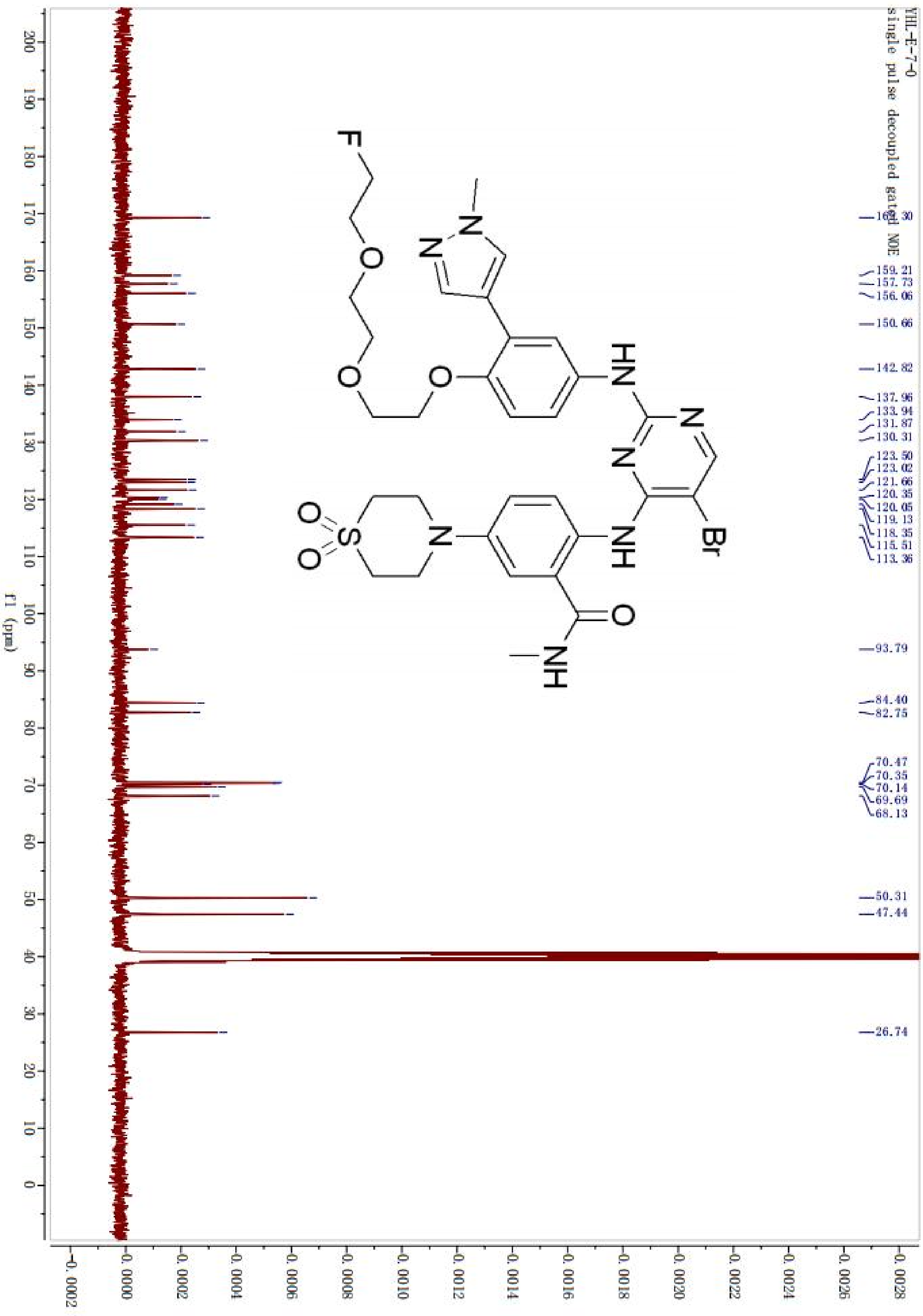

Figure S8 ^{13}C NMR spectrum of **7c**

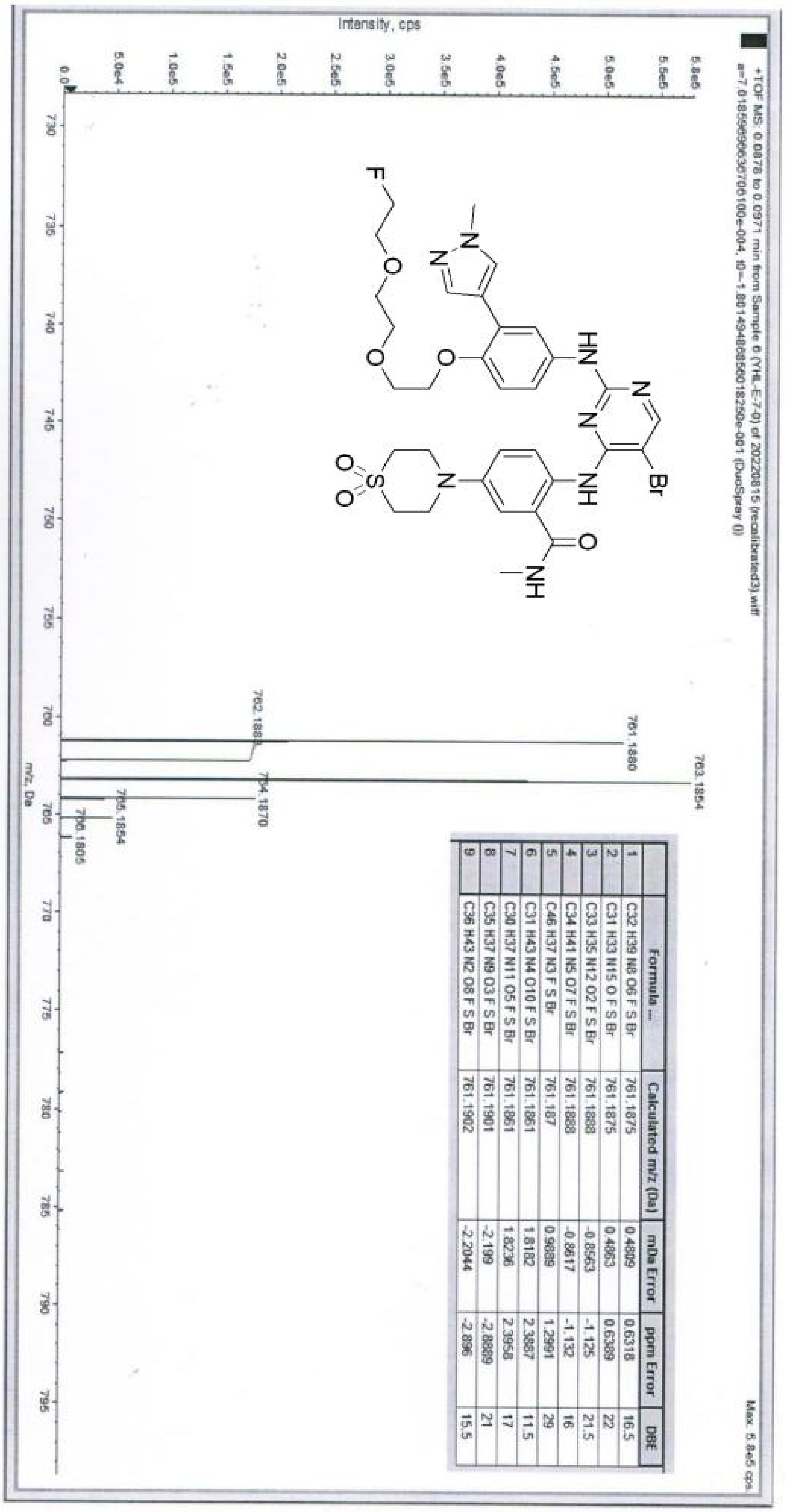

	Formula ...	Calculated m/z (Da)	mDa Error	ppm Error	DBE
1	C32 H39 N8 O6 F S Br	761.1875	0.4809	0.6318	16.5
2	C31 H33 N15 O F S Br	761.1875	0.4863	0.6389	22
3	C33 H35 N12 O2 F S Br	761.1888	-0.8563	-1.125	21.5
4	C34 H41 N5 O7 F S Br	761.1888	-0.8617	-1.132	16
5	C46 H37 N3 F S Br	761.187	0.9689	1.2991	29
6	C31 H43 N4 O10 F S Br	761.1861	1.8182	2.3887	11.5
7	C30 H37 N11 O5 F S Br	761.1861	1.8236	2.3958	17
8	C35 H37 N9 O3 F S Br	761.1901	-2.199	-2.8889	21
9	C36 H43 N2 O8 F S Br	761.1902	-2.2044	-2.896	15.5

Figure S9 ESI-HRMS spectrum of **7c**

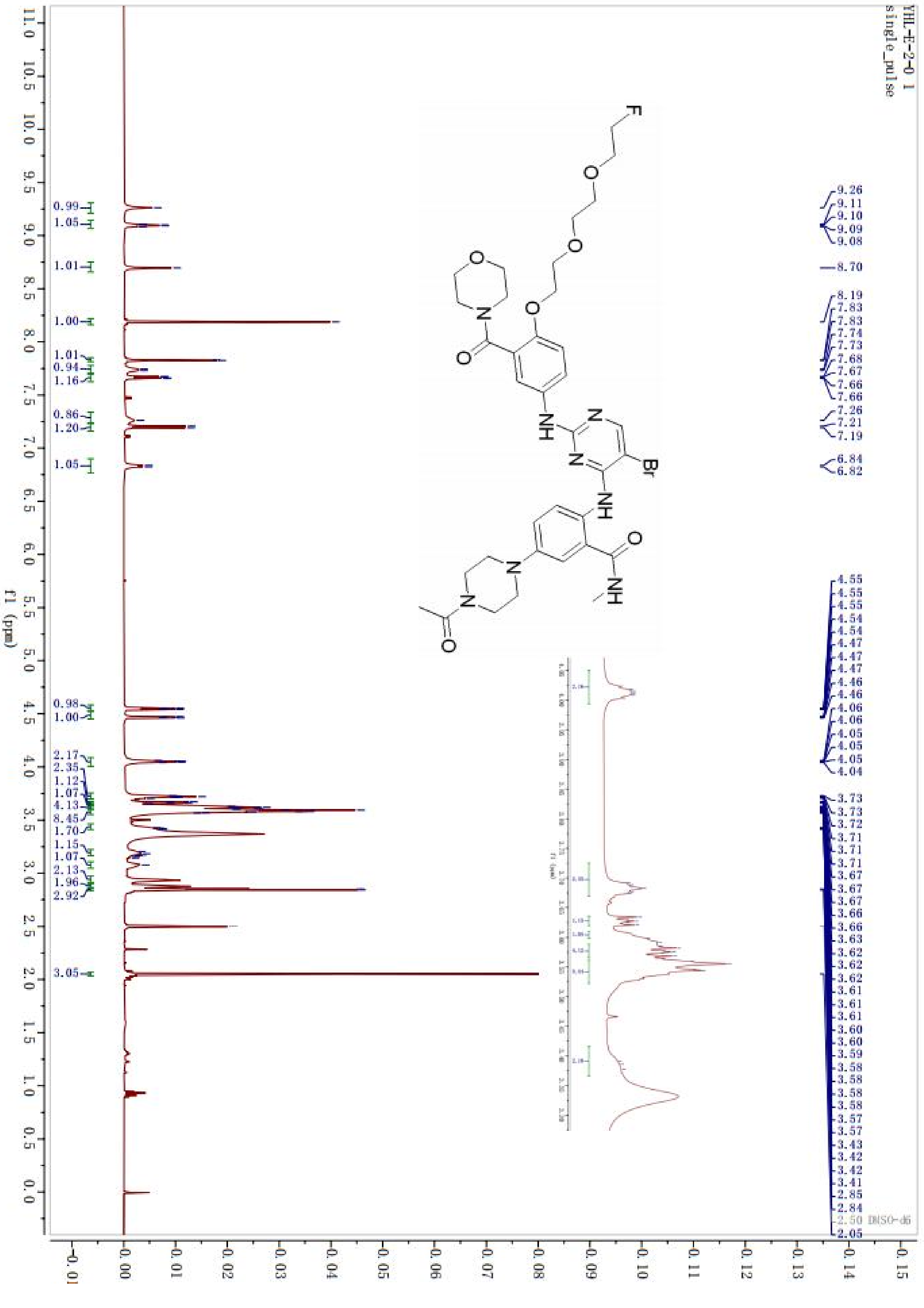

Figure S10 ^{1}H NMR spectrum of **7d**

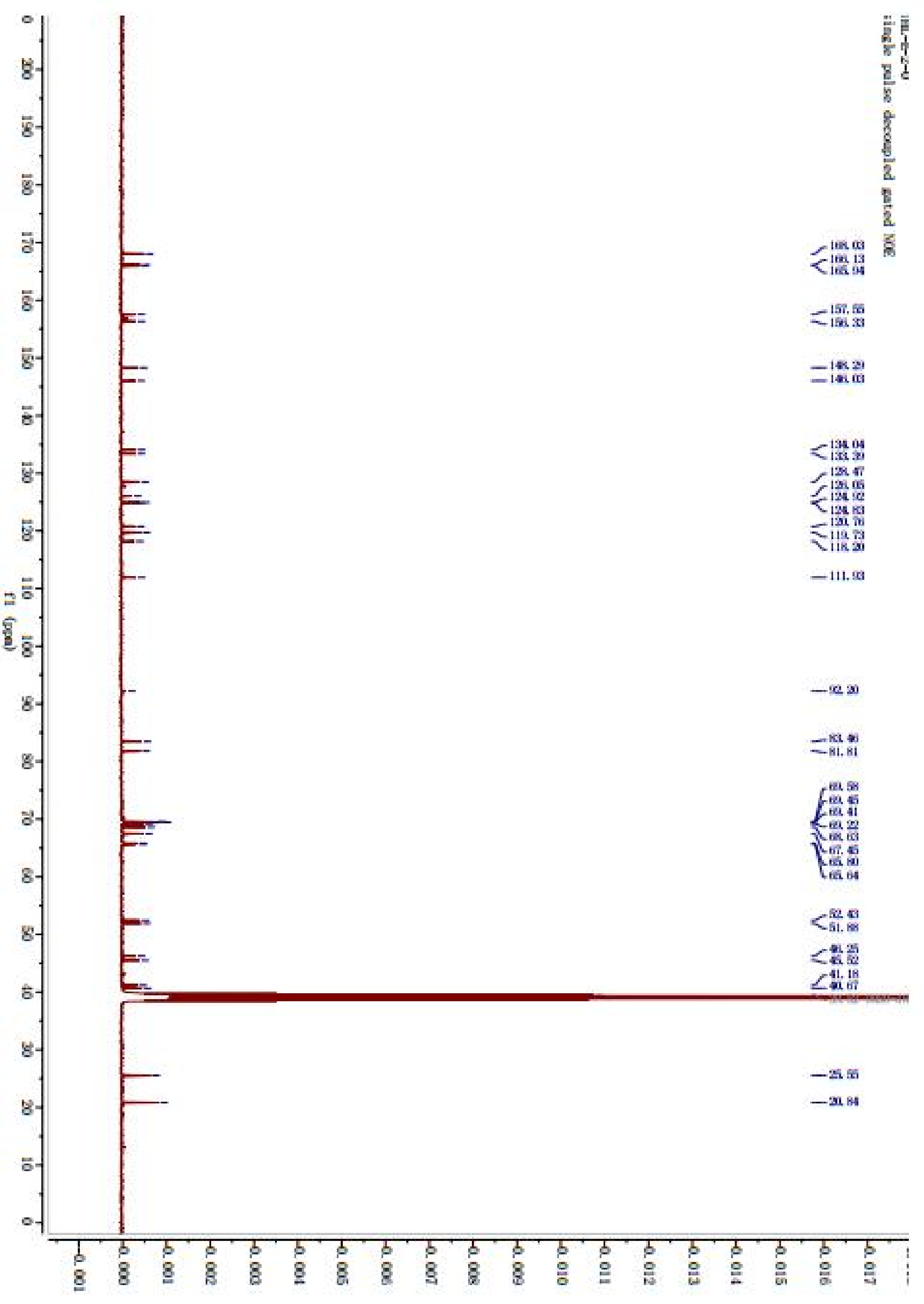

Figure S11 ^{13}C NMR spectrum of **7d**

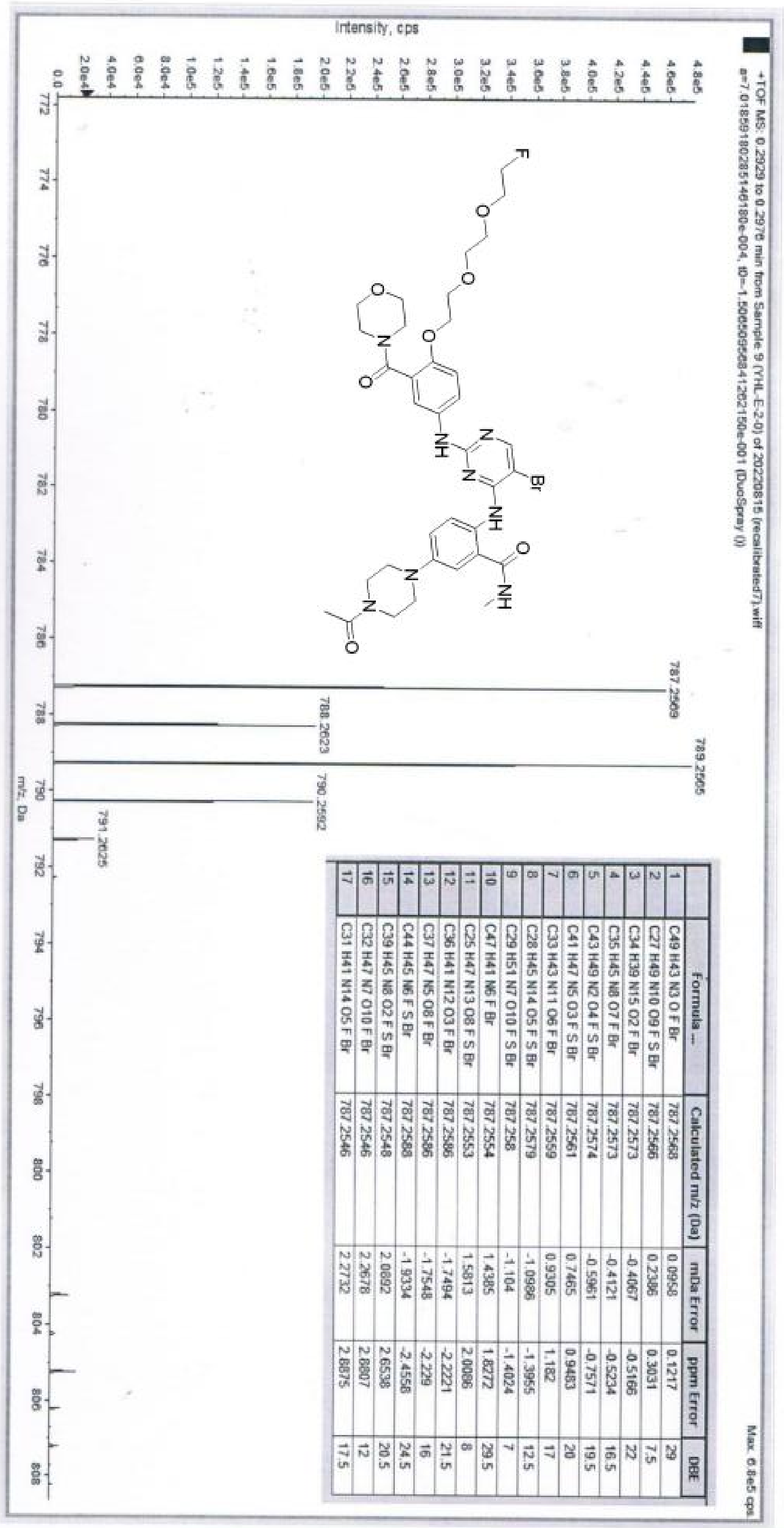

	Formula ...	Calculated m/z (Da)	mDa Error	ppm Error	DBE
1	C49 H43 N3 O F Br	787.2568	0.0958	0.1217	29
2	C27 H49 N10 O9 F S Br	787.2566	0.2386	0.3031	7.5
3	C34 H39 N15 O2 F Br	787.2573	-0.4067	-0.5166	22
4	C35 H45 N8 O7 F Br	787.2573	-0.4121	-0.5234	16.5
5	C43 H49 N2 O4 F S Br	787.2574	-0.5961	-0.7571	19.5
6	C41 H47 N5 O3 F S Br	787.2561	0.7465	0.9483	20
7	C33 H43 N11 O6 F Br	787.2559	0.9305	1.182	17
8	C28 H45 N14 O5 F S Br	787.2579	-1.0986	-1.3955	12.5
9	C29 H51 N7 O10 F S Br	787.258	-1.104	-1.4024	7
10	C47 H41 N6 F Br	787.2554	1.4385	1.8272	29.5
11	C25 H47 N13 O8 F S Br	787.2553	1.5813	2.0086	8
12	C36 H41 N12 O3 F Br	787.2586	-1.7494	-2.2221	21.5
13	C37 H47 N5 O8 F Br	787.2586	-1.7548	-2.229	16
14	C44 H45 N6 F S Br	787.2588	-1.9334	-2.4558	24.5
15	C39 H45 N8 O2 F S Br	787.2548	2.0892	2.6538	20.5
16	C32 H47 N7 O10 F Br	787.2546	2.2678	2.8807	12
17	C31 H41 N14 O5 F Br	787.2546	2.2732	2.8875	17.5

Figure S12 ESI-HRMS spectrum of **7d**

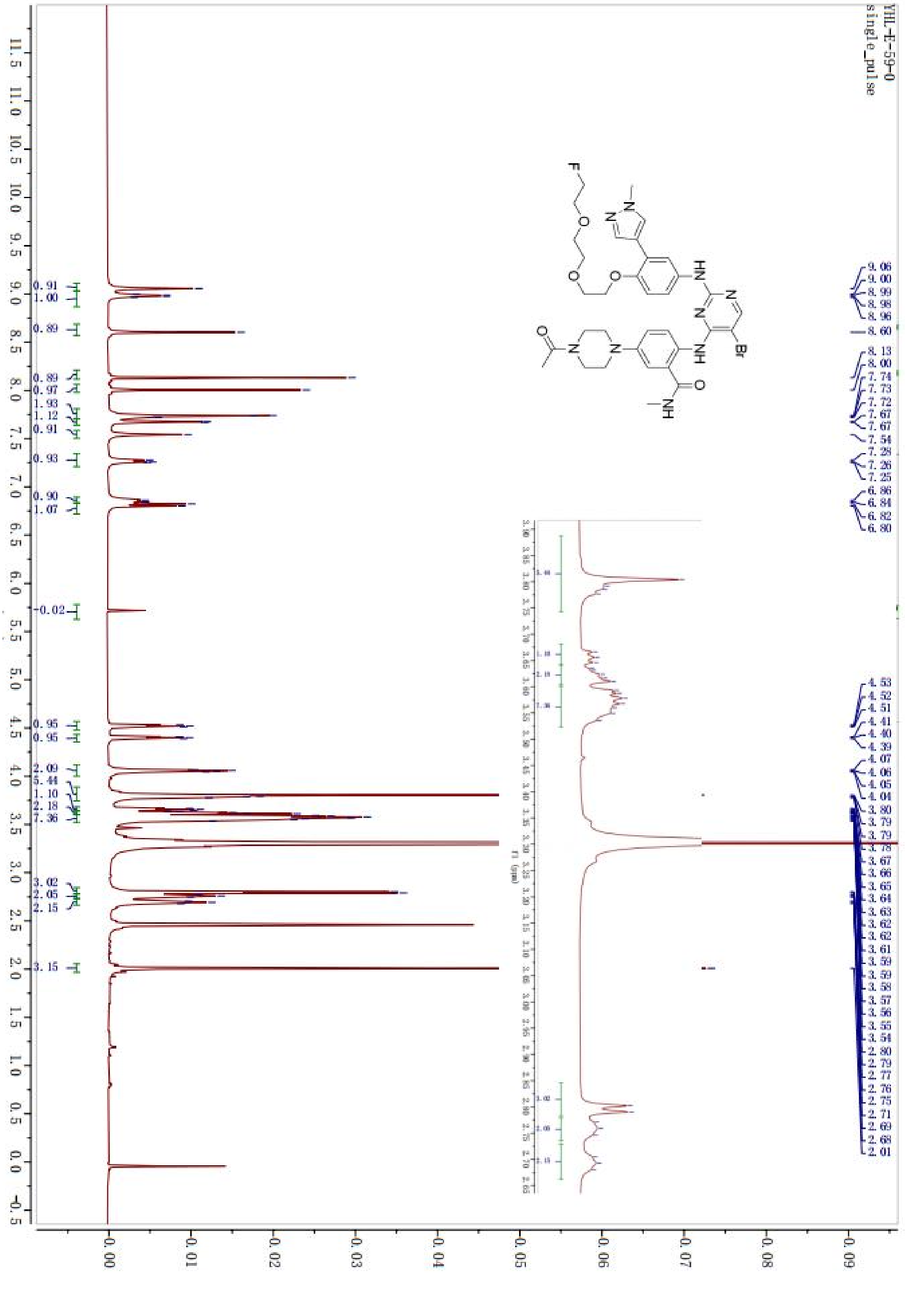

Figure S13 ^{1}H NMR spectrum of **7e**

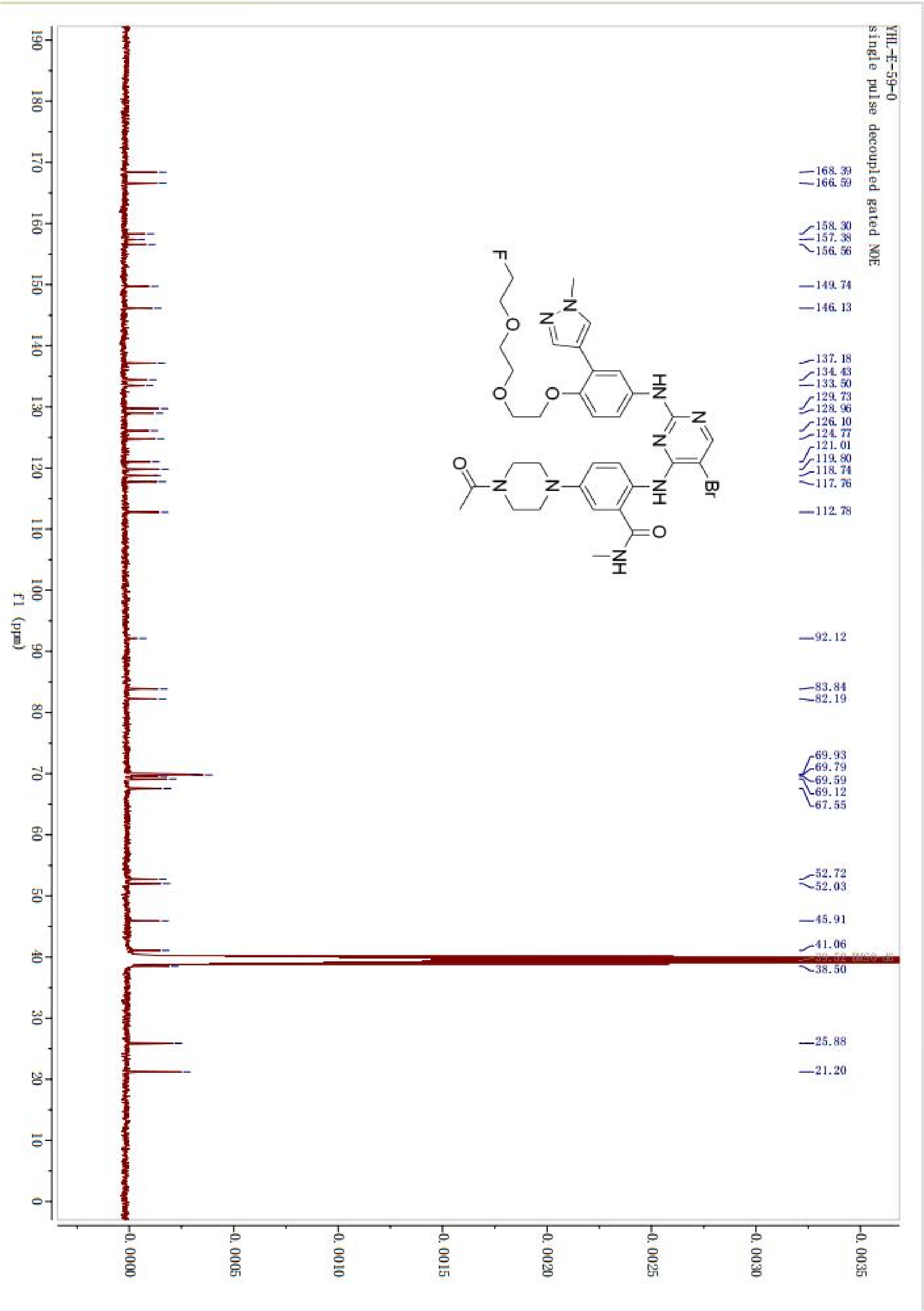

Figure S14 ^{13}C NMR spectrum of **7e**

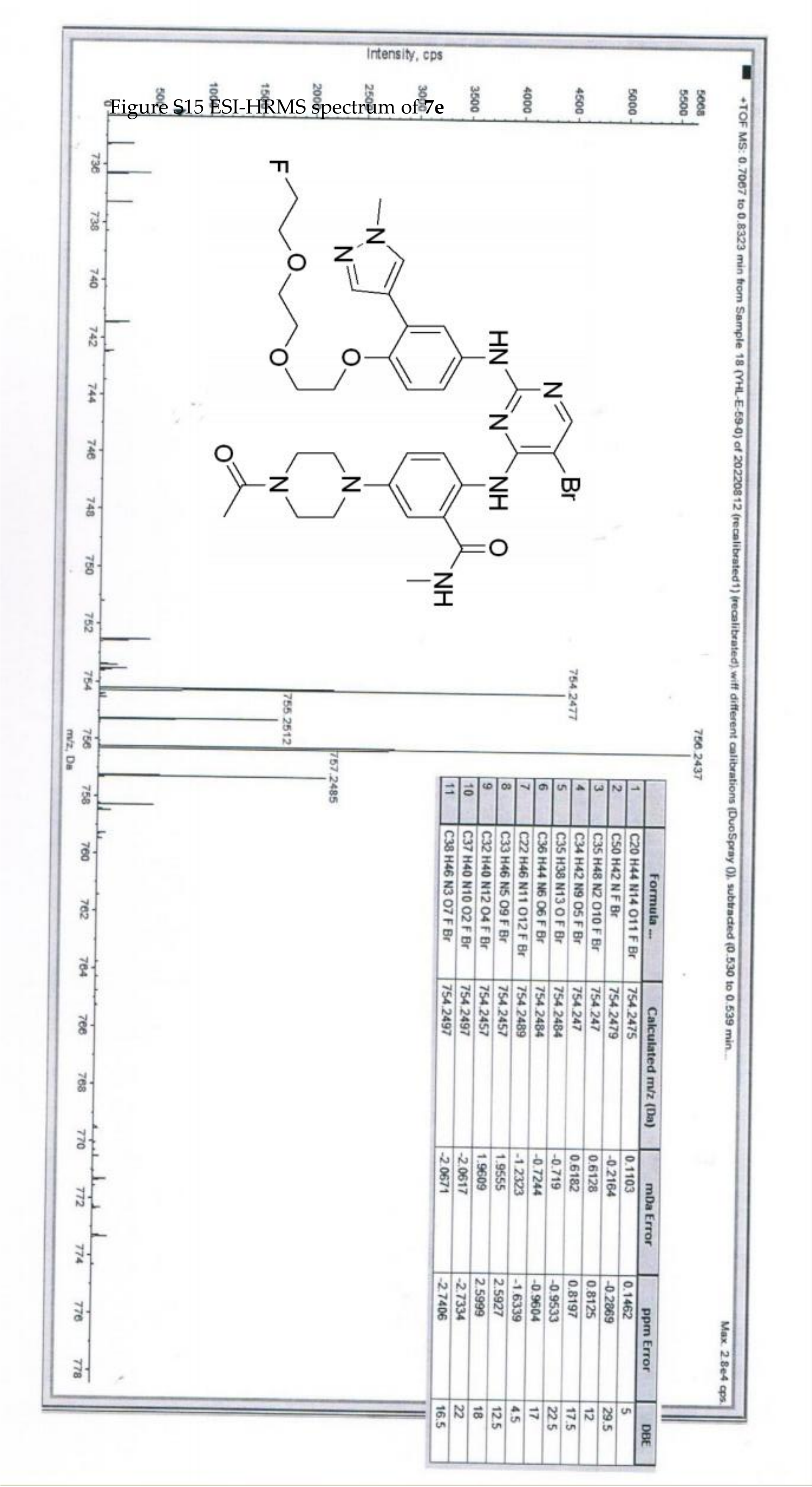

	Formula ...	Calculated m/z (Da)	mDa Error	ppm Error	DBE
1	C20 H44 N14 O11 F Br	754.2475	0.1103	0.1462	5
2	C50 H42 N F Br	754.2479	-0.2164	-0.2869	29.5
3	C35 H48 N2 O10 F Br	754.247	0.6128	0.8125	12
4	C34 H42 N9 O5 F Br	754.247	0.6182	0.8197	17.5
5	C35 H38 N13 O F Br	754.2484	-0.719	-0.9533	22.5
6	C36 H44 N6 O6 F Br	754.2484	-0.7244	-0.9604	17
7	C22 H46 N11 O12 F Br	754.2489	-1.2323	-1.6339	4.5
8	C33 H46 N5 O9 F Br	754.2457	1.9555	2.5927	12.5
9	C32 H40 N12 O4 F Br	754.2457	1.9609	2.5999	18
10	C37 H40 N10 O2 F Br	754.2497	-2.0617	-2.7334	22
11	C38 H46 N3 O7 F Br	754.2497	-2.0671	-2.7406	16.5

Figure S15 ESI-HRMS spectrum of **7e**

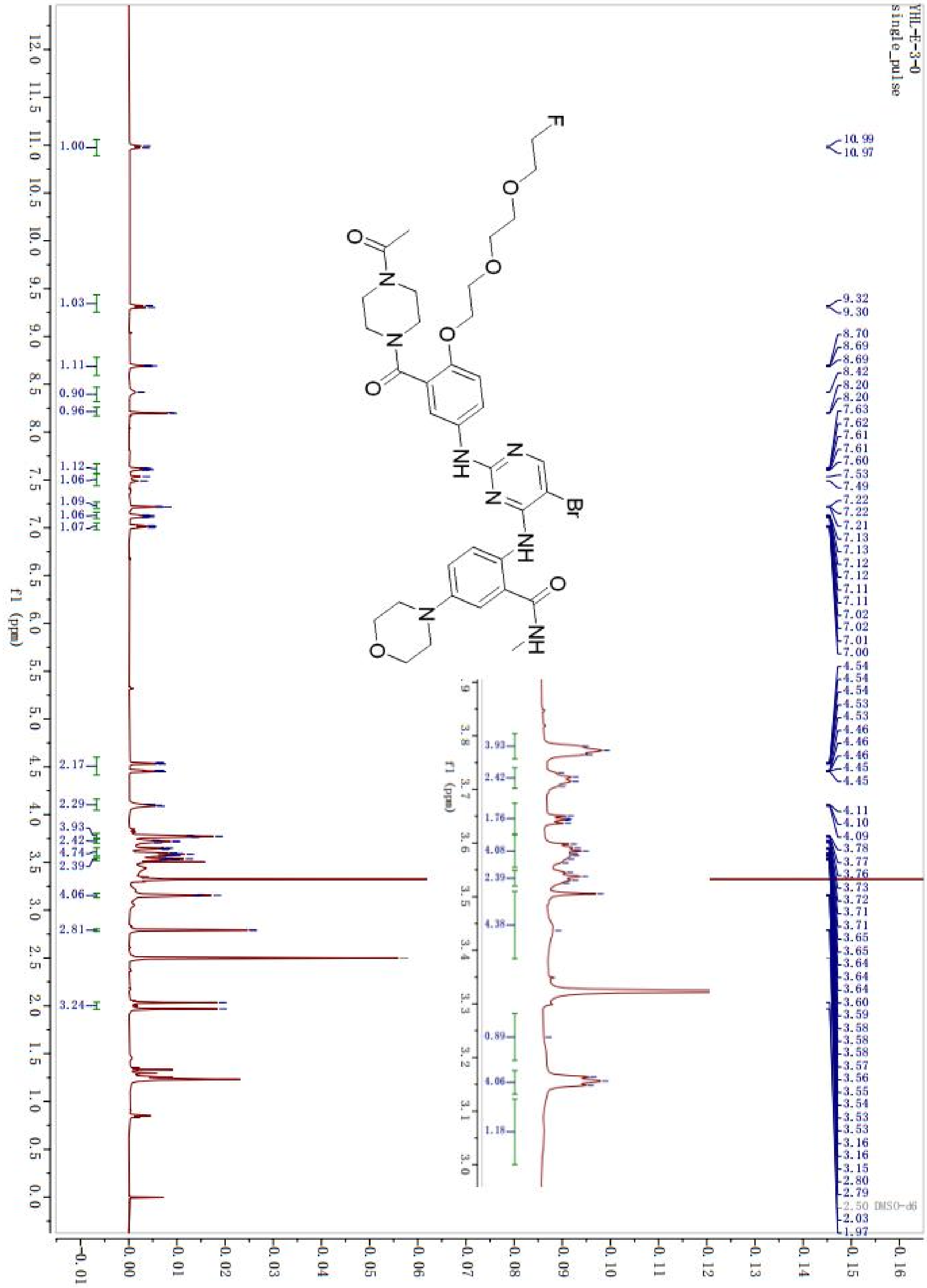

Figure S16 ^{1}H NMR spectrum of **7f**

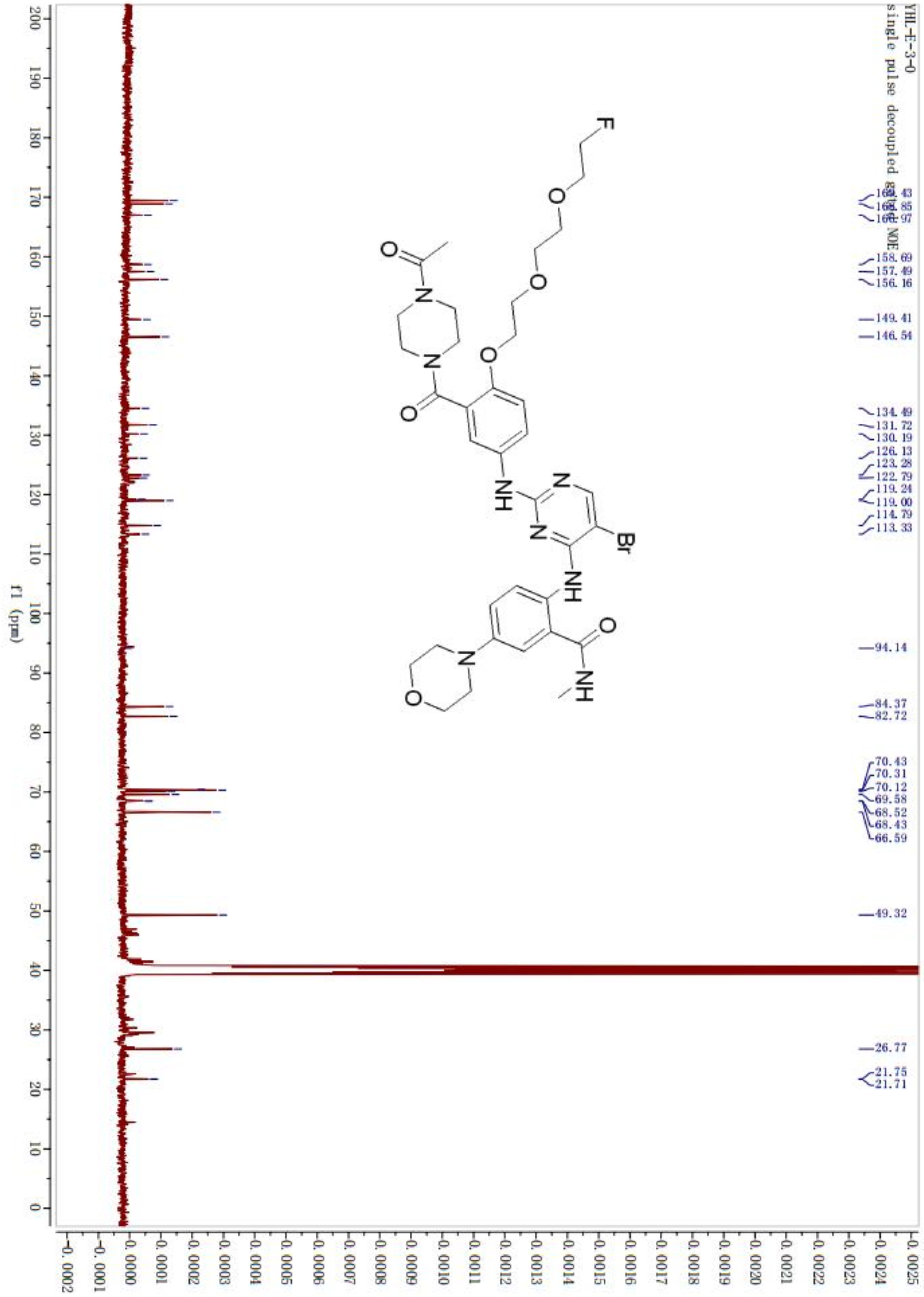

Figure S17 ^{13}C NMR spectrum of **7f**

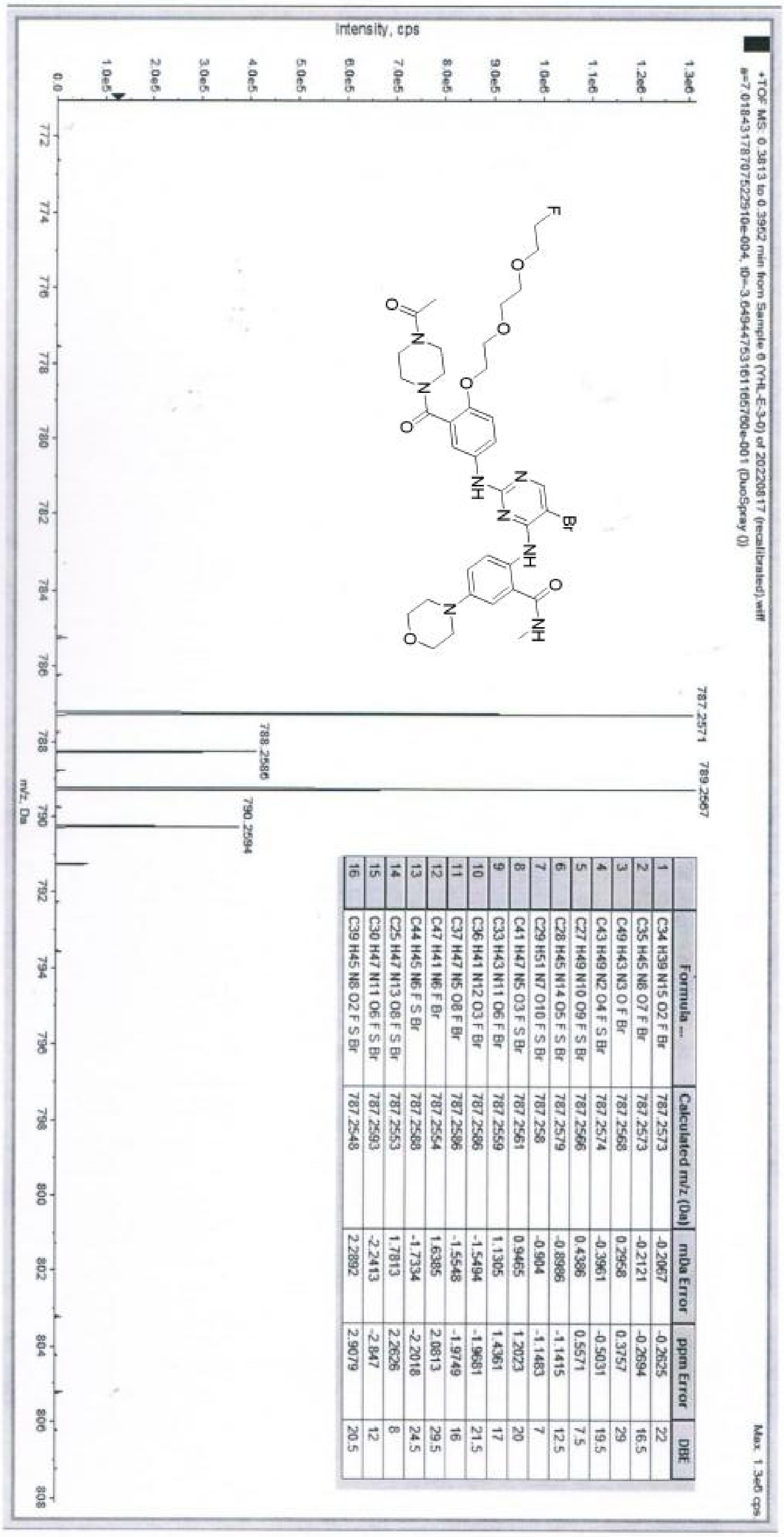

Figure S18 ESI-HRMS spectrum of **7f**

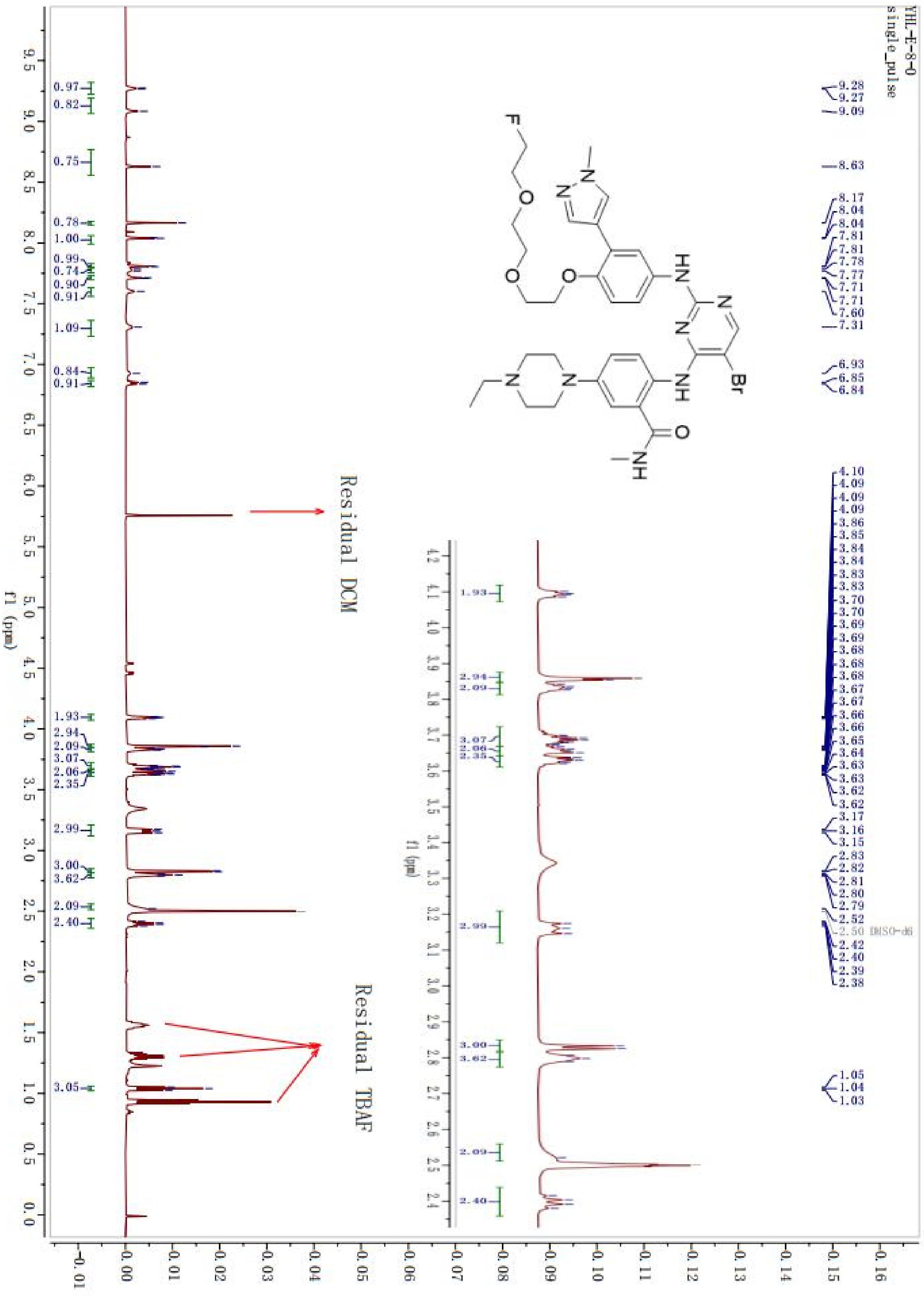

Figure S19 ^{1}H NMR spectrum of **7g**

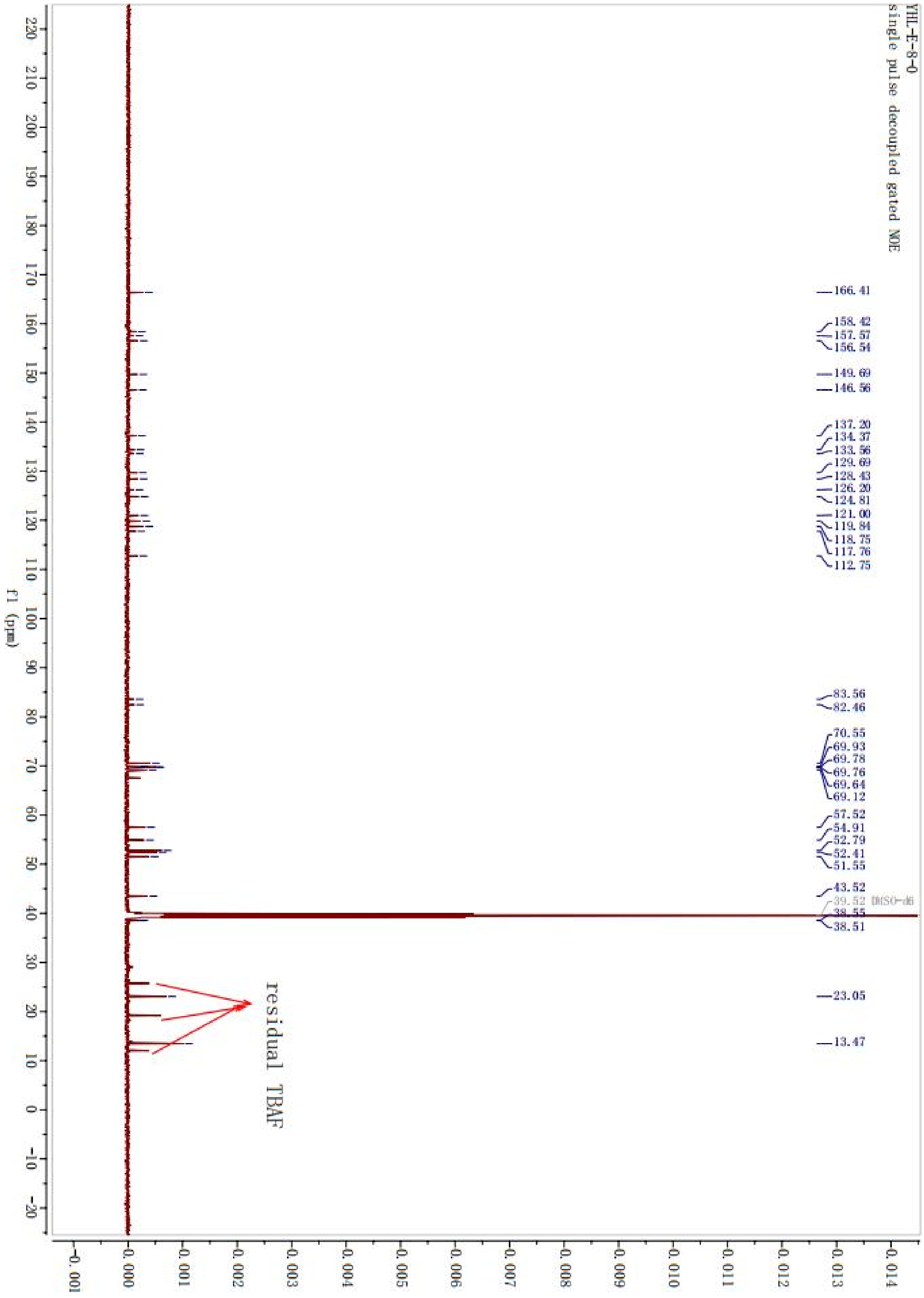

Figure S20 ^{13}C NMR spectrum of **7g**

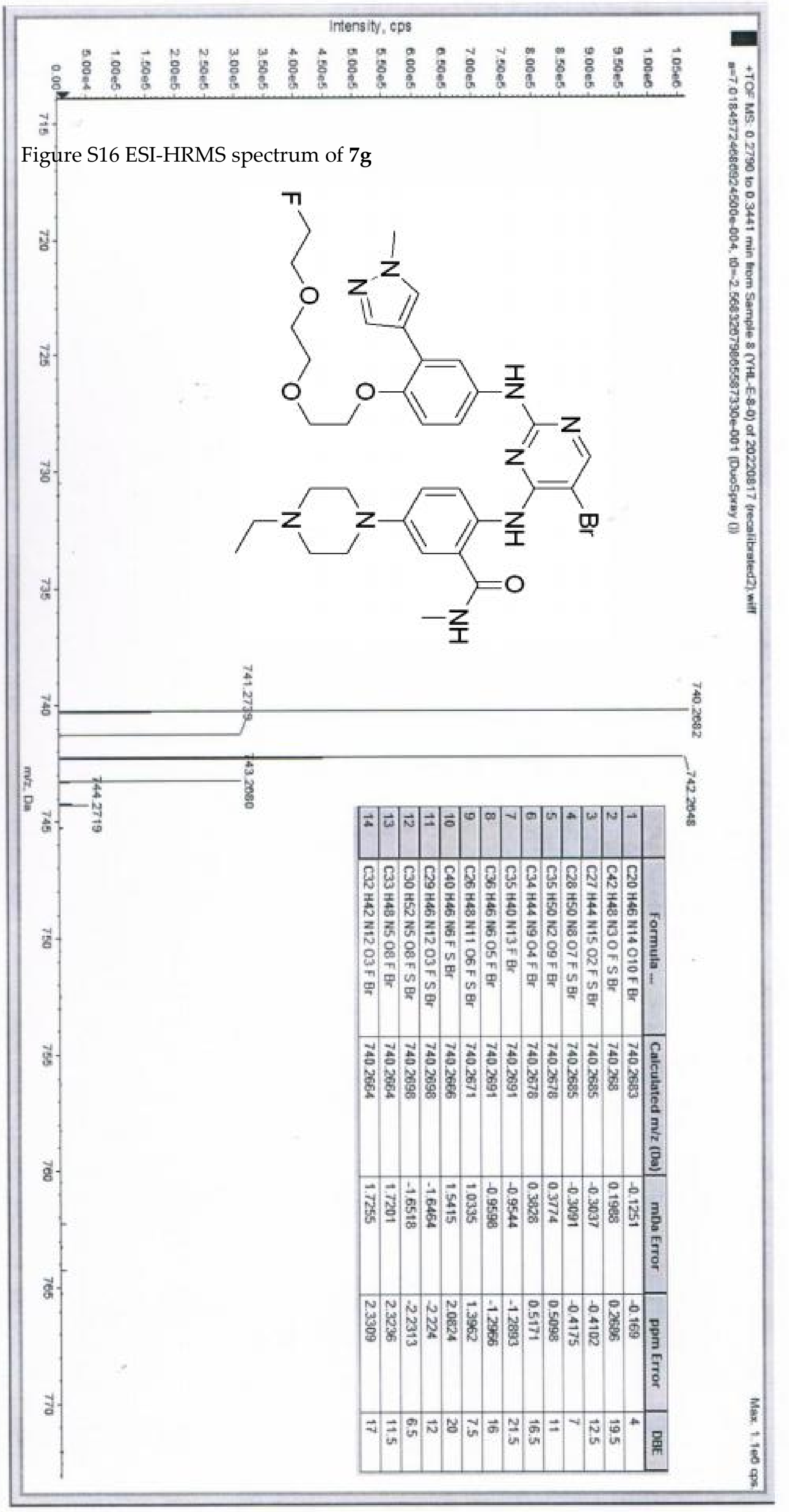

Figure S16 ESI-HRMS spectrum of **7g**

Figure S21 ESI-HRMS spectrum of **7g**
